# Investigation of exJSRV LTR promoter activity based on transcription factor regulatory networks

**DOI:** 10.3389/fvets.2025.1727983

**Published:** 2026-01-09

**Authors:** Xiaoyue Du, Pei Zhang, Xujie Duan, Anyu Bao, Xinqi Ma, Yufei Zhang, Shuying Liu

**Affiliations:** 1 College of Veterinary Medicine, Inner Mongolia Agricultural University, Hohhot, China; 2 Key Laboratory of Veterinary Fundamentals and Disease Prevention and Control for Herbivorous Livestock, Hohhot, Inner Mongolia, China; 3 Key Laboratory of Clinical Diagnosis and Treatment Technology in Animal Disease, Ministry of Agriculture, Hohhot, China

**Keywords:** exJSRV LTR, GATA3, MEK/ERK signaling pathway, oncogenic potential, transcription factors

## Abstract

Ovine pulmonary adenocarcinoma (OPA) is a contagious respiratory tumor affecting sheep and goats, caused by the Jaagsiekte sheep retrovirus (exJSRV). The transcriptional regulation of exJSRV is primarily governed by its long terminal repeat (LTR) region, which interacts with host transcription factors. However, the specific host factors and signaling pathways that modulate exJSRV LTR activity remain poorly understood. To address this knowledge gap, we combined bioinformatic prediction with *in vivo* and *in vivo* experiments to identify and validate host transcription factors involved in regulating exJSRV LTR activity. Analyses using the hTFtarget and AnimalTFDB databases identified 53 potential transcription factors interacting with the exJSRV LTR. KEGG enrichment analysis revealed that these factors were mainly associated with the MAPK signaling pathway, particularly the MEK/ERK branch. Activation of this pathway with C16-PAF significantly enhanced exJSRV LTR-driven transcription, while inhibition with U0126 reduced it, indicating a positive regulatory role. Among 18 candidate transcription factors examined, GATA3 exerted the most pronounced effect on transcriptional activity. Overexpression of GATA3 increased both LTR activity and Env protein expression, while GATA3 knockdown reduced them. *In vivo*, GATA3 overexpression promoted LTR-Env–induced tumor formation in nude mice, whereas GATA3 interference suppressed tumor growth. Furthermore, strong luciferase expression was detected in the lungs and livers of C57BL/6 mice infected with LV-exJSRV LTR-Nanoluc. In conclusion, this study demonstrates that both GATA3 and the MEK/ERK signaling pathway regulate exJSRV LTR activity. Additionally, the exJSRV LTR exhibited potential tendency in the lung and liver tissues of C57BL/6 pairs of mice. These findings provide new insights into the molecular mechanisms underlying OPA pathogenesis.

## 1 Introduction

Ovine pulmonary adenocarcinoma (OPA) is an acute and contagious respiratory disease affecting sheep and goats, caused by the exogenous β-retrovirus Jaagsiekte sheep retrovirus (exJSRV). The disease is characterized by dyspnea, coughing, progressive weight loss, and profuse serous nasal discharge, accompanied by neoplastic proliferation of type II pneumocytes and non-ciliated bronchiolar epithelial cells (1). Since its first description in South Africa in the 19th century, OPA has been reported across Europe, North and South America, and Asia (1–3). In China, the first outbreak was documented in 1951, and since then, OPA has remained an important cause of economic loss in sheep-farming regions.

The oncogenic mechanism of JSRV primarily depends on tissue-specific viral expression, which is governed by the viral envelope (Env) protein and its long terminal repeat (LTR) regulatory elements (4–7). The Env gene encodes a viral glycoprotein that interacts with specific host cell receptors such as Hyaluronidase-2 (HYAL2) to mediate viral entry (8). Thus, host receptor distribution determines the cellular tropism of the virus. Meanwhile, the LTR region, which contains the viral promoter and enhancer sequences, plays a pivotal role in regulating viral transcription and determining tissue specificity after integration into the host genome (9, 10).

Recent studies have revealed that the exJSRV exhibits cell-type-dependent promoter activity, with strong transcriptional activity in alveolar cells (11), consistent with the tissue tropism observed in OPA lesions (1, 12). The U3 region of the LTR contains several putative transcription factor binding motifs, including those for HNF-3/FOXA2, AP-1, NF-κB, and GATA family proteins, which are involved in epithelial differentiation and inflammation. The regulatory complexity of the LTR enables exJSRV to hijack host transcriptional machinery and respond to cellular signaling pathways, such as MAPK/ERK and PI3K/AKT, which are frequently activated in epithelial tumorigenesis (13, 14).

In this study, we identified that the upstream site (−146/−132) of the exJSRV LTR and the MEK/ERK pathway play crucial roles in regulating LTR activity. Among the 18 transcription factors predicted to bind this region, GATA3 exhibited the strongest activation of LTR-driven transcription. Moreover, GATA3 overexpression promoted LTR-Env-mediated tumor formation, whereas GATA3 knockdown suppressed it. These findings suggest that GATA3 cooperates with the MEK/ERK pathway to enhance exJSRV transcription and tumorigenesis, providing novel insights into the molecular mechanisms underlying OPA pathogenesis.

## 2 Materials and methods

### 2.1 Cells and animals

OAR-L1 (ovine alveolar type II epithelial cells), 293T (human embryonic kidney cells), and BEAS-2B (human bronchial epithelial cells) cell lines were obtained from the China Center for Type Culture Collection (CCTCC). All cells were cultured in Dulbecco’s Modified Eagle Medium (DMEM; Gibco, United States) supplemented with 10% fetal bovine serum (FBS; Gibco) and 1% penicillin–streptomycin at 37 °C in a humidified incubator containing 5% CO_2_. Five-week-old specific pathogen-free (SPF) BALB/C-nu/nu nude mice and six-week-old male C57BL/6 mice were purchased from Beijing Weitonglihua Experimental Animal Co., Ltd. All animal procedures were conducted according to institutional ethical guidelines and approved by the Animal Ethics Committee of Sichuan Agricultural University.

### 2.2 Mapping of exJSRV LTR regulatory elements

To identify potential transcription factors (TFs) binding to the exJSRV long terminal repeat (LTR), we submitted its full-length sequence (Supplementary Table S1) to the hTFtarget^1^ and AnimalTFDB v4.0^2^ databases. The resulting predictions were screened using a significance threshold of *p* < 0.05. The exJSRV LTR sequence is shown in Supplementary Table S1. The full-length exJSRV LTR sequence was amplified by PCR and inserted into the pGL4.10 vector between the *KpnI* and *HindIII* restriction sites. The construct, named pGL4.10/exJSRV LTR, was confirmed by restriction enzyme digestion and Sanger sequencing. Two specific LTR regions were selected for mutagenesis: the upstream site (−146 to −132, designated T1) and the downstream site (−47 to −32, designated T2). Site-directed mutagenesis was performed using homologous recombination with the pGL4.10/exJSRV LTR plasmid as a template, generating the mutant constructs pGL4.10/exJSRV LTR-T1 and pGL4.10/exJSRV LTR-T2. All mutations were verified by restriction enzyme digestion and sequencing. OAR-L1 cells were transfected with either the wild-type or mutant reporter plasmids using Lipofectamine 3000 (Invitrogen, United States). After 24 h, LTR-driven transcriptional activity was assessed using a Dual-Luciferase Reporter Assay System (Promega, United States) following the manufacturer’s protocol.

### 2.3 MEK/ERK pathway modulation

We performed KEGG pathway enrichment analysis on the predicted exJSRV LTR-binding transcription factors via the DAVID database. Using a threshold of *p* < 0.05, we identified significantly enriched signaling pathways and selected the MEK/ERK signaling pathway from among these for experimental verification. OAR-L1 cells at 60%–70% confluence were co-transfected with 2 μg of pGL4.10/exJSRV LTR and 50 ng of the control vector pGL4.74. Eight hours post-transfection, the medium was replaced, and cells were treated with one of the following: blank control, vehicle control (0.1% DMSO), 1 μM C16-PAF (MEK/ERK activator), or 3 μM U0126 (MEK/ERK inhibitor). After 24 h, total protein was extracted for Western blotting to confirm pathway activation, and luciferase activity was quantified.

### 2.4 Functional screening of transcription factors binding the −146/−132 region

Eighteen transcription factors predicted to interact with the −146/−132 region were selected for screening. Each gene was cloned into a eukaryotic expression vector, and successful cloning was confirmed by qPCR (primer sequences are listed in Supplementary Table S1). Protein–DNA binding was examined using electrophoretic mobility shift assays (EMSA). Three probes were used: an IR680-labeled wild-type probe (T1-Wt-IR680), an unlabeled wild-type competitor (T1-Wt), and an unlabeled mutant competitor (T1-Mut). All probe sequences are provided in Supplementary Table S2. Binding reactions were incubated at room temperature for 30 min and analyzed on a 6% native polyacrylamide gel using a fluorescence imaging system.

### 2.5 Role of GATA3 in exJSRV LTR-env oncogenic potential

A lentiviral vector carrying the exJSRV LTR-env construct was produced in 293 T cells using a standard packaging system (pGag/Pol, pREV, and pVSV-G). The viral supernatant was harvested 48 h after transfection, filtered, and used to transduce BEAS-2B cells. Stable LTR-env cell lines were established by selection with 0.3 μg/mL puromycin for 7 d. The stable cells were divided into four groups: LTR–env only, empty-vector control, GATA3 overexpression (transfected with p3xFLAG-CMV-14/GATA3), and GATA3 knockdown (transfected with pGPU6/GFP/Neo-shGATA3). After 48 h, total protein was extracted and Env expression was detected by Western blot. For the *in vivo* tumorigenicity assay, 3 × 10^7^ cells suspended in 1 mL PBS were injected subcutaneously into the right flank of nude mice (*n* = 8 per group). Tumor volume was measured weekly using the formula (length × width^2^)/2. On day 32, mice were euthanized, and tumors were excised, weighed, and collected for histological analysis.

### 2.6 Investigation of exJSRV LTR tissue tropism

To assess the tissue tropism of the exJSRV LTR, a recombinant plasmid, pLVX-exJSRV LTR-Nanoluc, was constructed. For lentiviral packaging (LV-exJSRV LTR-Nanoluc), 293 T cells were co-transfected with 100 μg of the expression plasmid and 100 μg of the packaging mix (pGag/Pol, pREV, pVSV-G) using polyethylenimine (PEI). After 48 h, the viral supernatant was collected, clarified by centrifugation (3,000 × *g*, 10 min), filtered (0.45 μm), and concentrated by ultracentrifugation at 25,000 rpm for 2 h.

OAR-L1, A549, and NIH3T3 cells at logarithmic growth phase were infected with the lentivirus at a multiplicity of infection (MOI) of 50 of LV-exJSRV LTR-Nanoluc in the presence of 8 μg/mL polybrene. After 12–16 h, the medium was replaced with fresh DMEM, and cells were cultured for 72 h before measuring Nanoluc luciferase activity using a commercial kit (Promega, United States). For *in vivo* tropism analysis, six-week-old male C57BL/6 mice were injected via the tail vein with 5 × 10^8^ viral genomes (vg) of LV-exJSRV LTR-Nanoluc. After 21 days, a substrate solution of Hydroxyfluorofurimazine (HFFz, 15 μg/mL) was administered intraperitoneally at 100 μL per 20 g body weight. Five to ten minutes later, mice were anesthetized with 2% isoflurane and imaged using an *in vivo* imaging system (IVIS Lumina III, PerkinElmer, United States). Luminescent signals were acquired under standardized exposure settings. After imaging, mice were placed in a warm environment until full recovery. All raw image data were archived for later analysis.

### 2.7 Statistical analysis

All statistical analyses were performed using GraphPad Prism 9.5 (GraphPad Software, United States). Data are presented as mean ± standard deviation (SD). Comparisons between two groups were conducted using unpaired Student *t*-tests, while multiple-group comparisons were analyzed using one-way analysis of variance (ANOVA). Differences were considered statistically significant when *p* < 0.05.

## 3 Results

### 3.1 Targeted mutation of the upstream FOXA2 motif (−146/−132) decreases LTR activity

**Figure 1:**
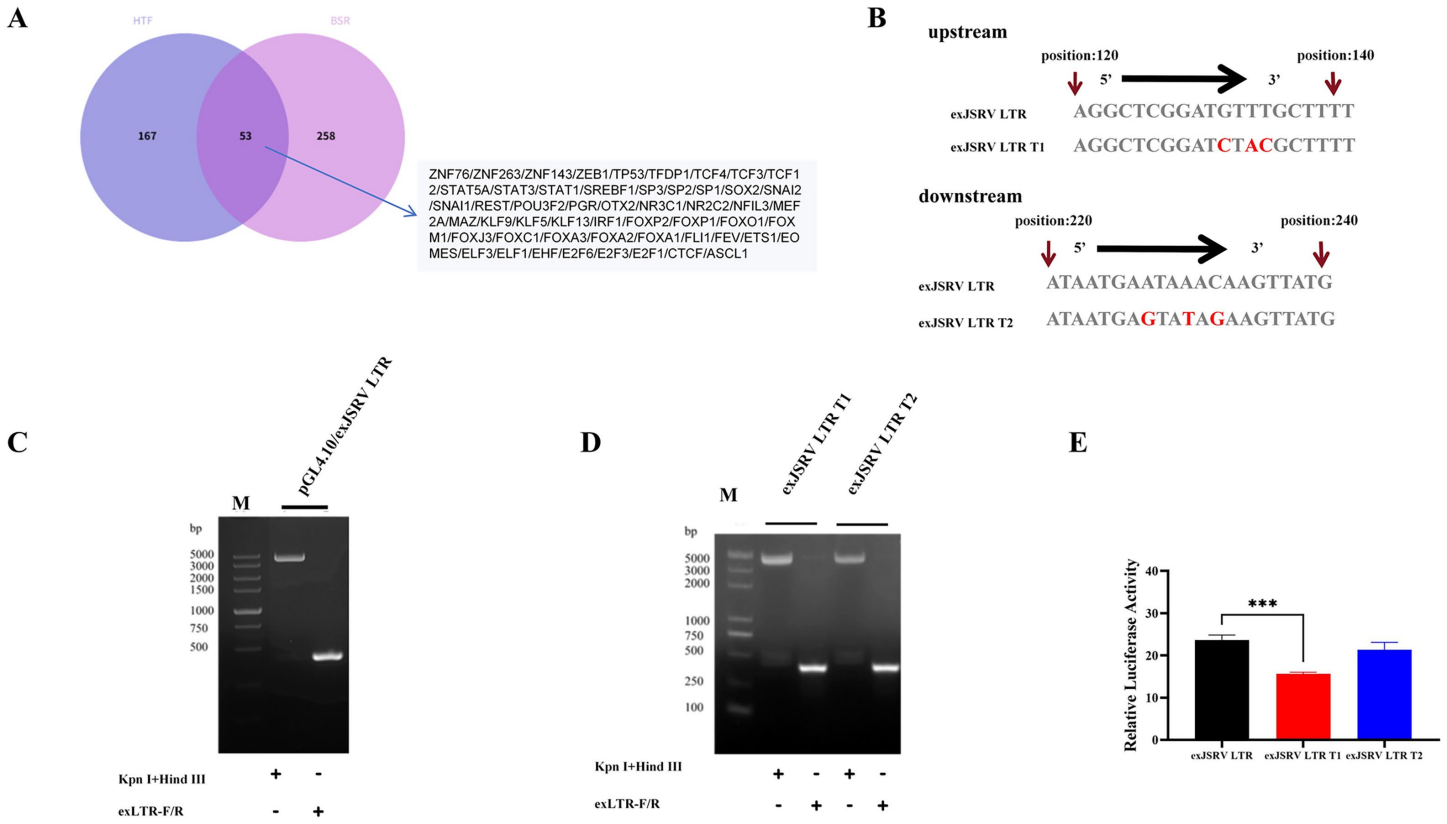
Analysis and identification of exJSRV LTR transcriptional regulatory elements. **(A)** Venn diagram identifying potential transcription factors of exJSRV LTR intersected by hTFtarget and AnimalTFDB v4.0. **(B)** Verification of the pGL4.10/exJSRV LTR luciferase reporter plasmid. **(C)** Schematic diagram of mutations at the upstream (−146 to −132) and downstream (−47 to −32) FOXA2 sites on exJSRV LTR. **(D)** Verification of the pGL4.10/exJSRV LTR T1 and T2 mutant plasmids. **(E)** The promoter activity of mutant exJSRV LTR plasmids was measured using a dual-luciferase reporter assay and is presented relative to the exJSRV LTR. Relative luciferase activity was calculated as the ratio of firefly to Renilla luciferase signal intensity. Data are shown as the mean ± SD from three independent replicates. ****p < 0.0001, ***p < 0.001, **p < 0.01, *p < 0.05. M: DL500 DNA marker.

From the hTFtarget and AnimalTFDB v4.0 databases, we identified 1,072 and 1,257 transcription factor (TF) binding sites associated with the exJSRV LTR, respectively, using a significance threshold of *p* < 0.05 (Supplementary Table S3). A Venn analysis of the TFs from these two datasets revealed 53 shared factors (Figure 1A). To validate these findings, we selected FOXA2 for further verification. The analysis indicated that the exJSRV LTR contains two adjacent consensus motifs for FOXA2, located at positions −146/−132 and −47/−32 (Supplementary Table S3). The pGL4.10/exJSRV LTR reporter plasmid was successfully constructed (Figure 1B) and further engineered to introduce multi-base substitutions at either the upstream (−146/−132) or downstream (−47/−32) FOXA2 binding sites, generating pGL4.10/exJSRV LTR-T1 and -T2, respectively (Figures 1C,D). Dual-luciferase reporter assays in OAR-L1 cells showed that mutation of the upstream site (T1) significantly reduced LTR-driven promoter activity by approximately 50% (*p* < 0.05), while mutation of the downstream site (T2) had no effect (*p* > 0.05) (Figure 1E). These results indicate that the −146/−132 region functions as a critical positive regulatory element for LTR transcriptional activity.

### 3.2 MEK/ERK pathway positively regulates exJSRV LTR promoter activity

**Figure 2:**
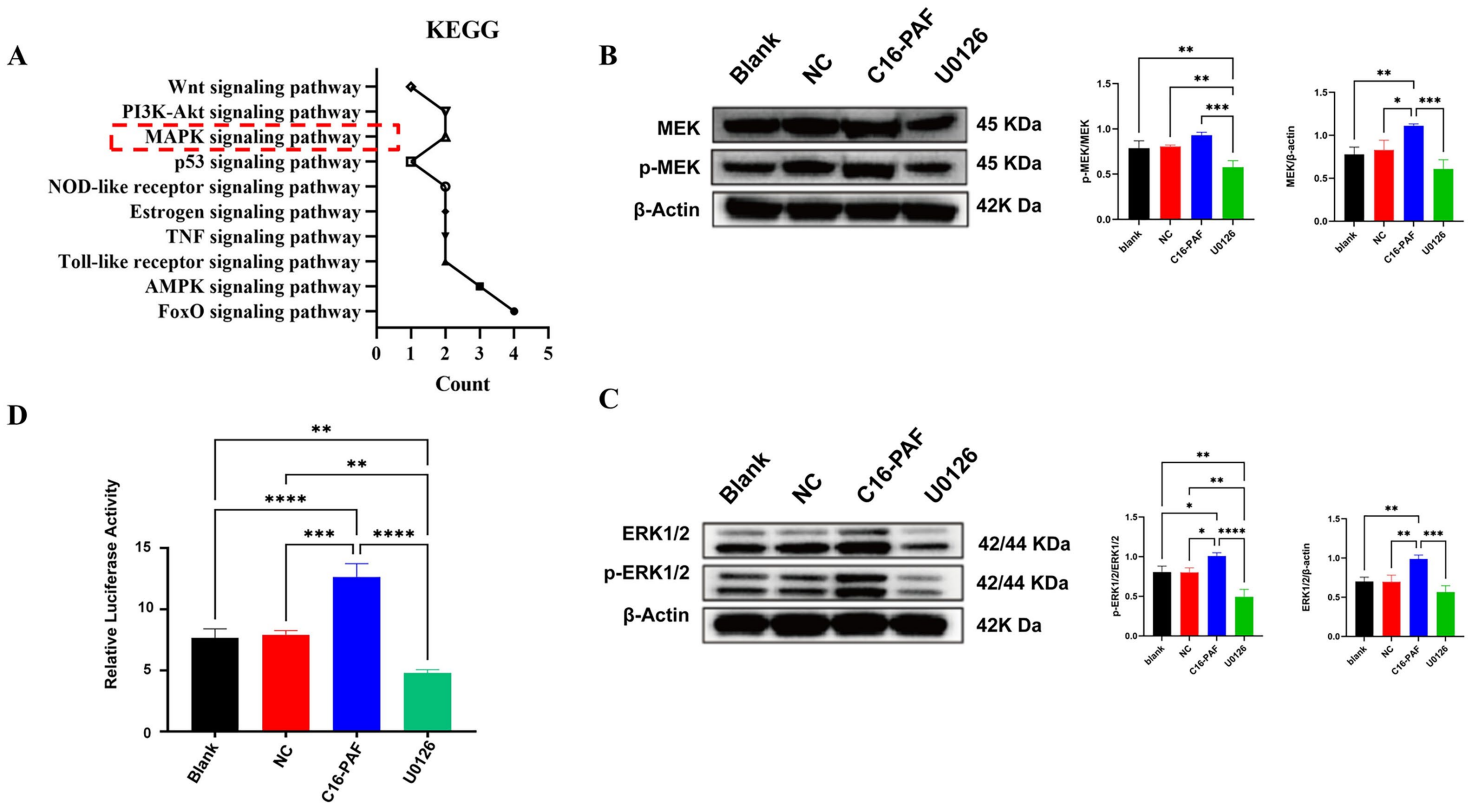
Impact of the MEK/ERK pathway on exJSRV LTR promoter activity. **(A)** KEGG enrichment analysis results. **(B)** Western blot validation of MEK pathway activation and inhibition. **(C)** Western blot validation of ERK pathway activation and inhibition. **(D)** Effect of MEK/ERK pathway activation and inhibition on exJSRV LTR promoter activity. Cells were treated as follows: Blank (untreated control), NC (vehicle control, 10% DMSO), C16-PAF (MEK/ERK activator), and U0126 (MEK/ERK inhibitor). Relative luciferase activity was calculated as the ratio of firefly to Renilla luciferase signal intensity. All values are mean ± SD of triplicate experiments. Data are from three independent experiments and presented as mean ± SD. ****p < 0.0001, ***p < 0.001, **p < 0.01, *p < 0.05.

KEGG pathway analysis of the predicted transcription factors showed enrichment in the MAPK, PI3K-AKT, and p53 signaling pathways (Figure 2A). The MEK/ERK pathway, a component of the MAPK cascade, was selected for experimental validation. Western blot analysis confirmed that treatment with the MEK/ERK activator C16-PAF markedly increased the phosphorylation levels of MEK and ERK1/2, whereas treatment with the inhibitor U0126 reduced their phosphorylation (Figures 2B,C). In dual-luciferase assays, activation of the MEK/ERK pathway significantly increased exJSRV LTR promoter activity, while inhibition of the pathway caused a notable decrease (Figure 2D). These findings demonstrate that the MEK/ERK pathway positively regulates the transcriptional activity of the exJSRV LTR.

### 3.3 GATA3 significantly enhances exJSRV LTR promoter activity

**Figure 3:**
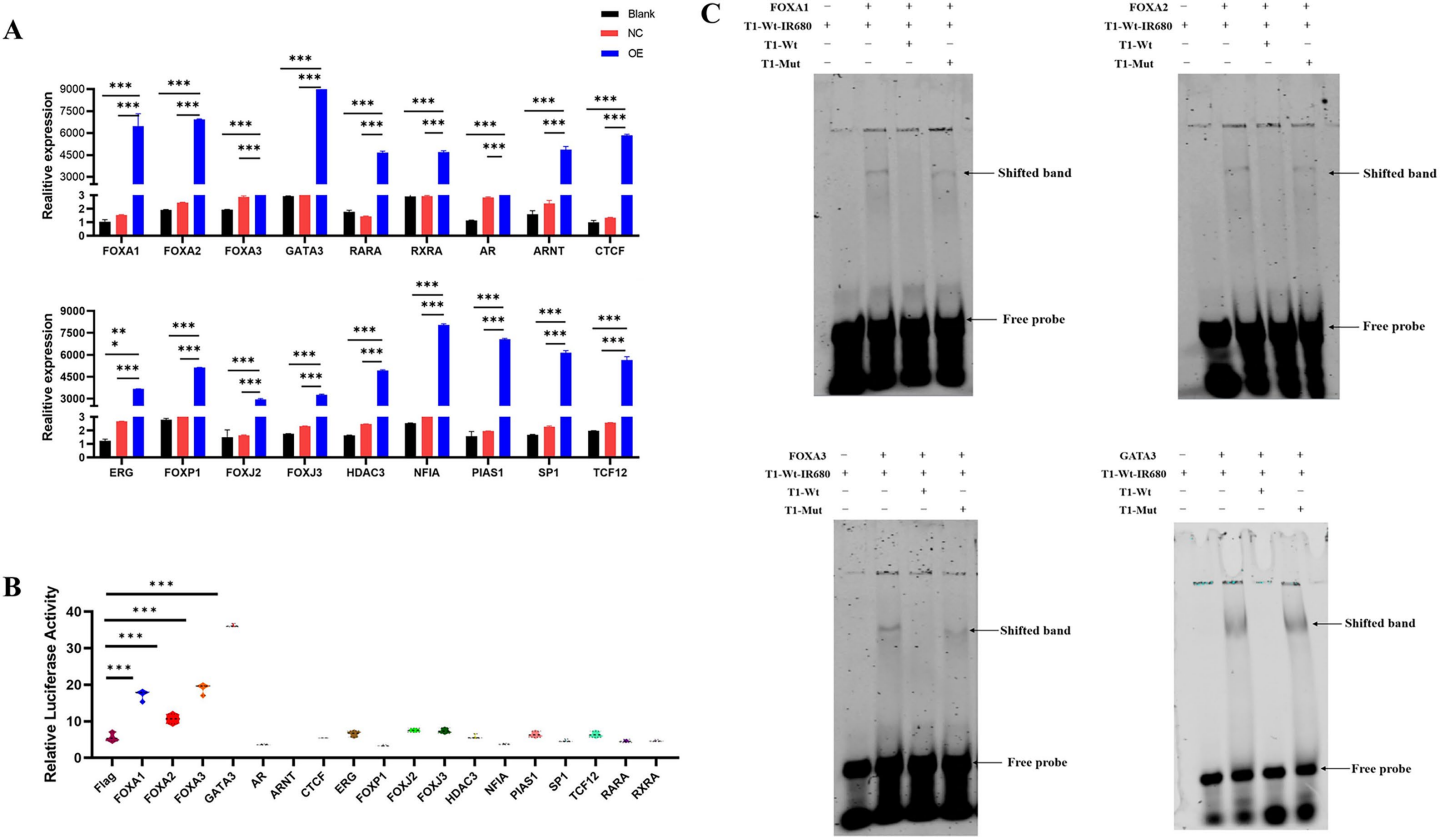
GATA3 significantly enhances exJSRV LTR promoter activity. **(A)** qPCR analysis verifying the overexpression efficiency of 18 candidate transcription factors in OAR-L1 cells. **(B)** The effect of overexpressing these 18 transcription factors on exJSRV LTR promoter activity, as determined by dual-luciferase reporter assay. **(C)** Electrophoretic mobility shift assay (EMSA) evaluating the binding of FOXA1, FOXA2, FOXA3, and GATA3 to the exJSRV LTR. The assay utilized an infrared dye-labeled wild-type probe (T1-WT-IR680). Binding specificity was confirmed by competition with unlabeled wild-type (T1-WT) and mutant (T1-Mut) probes. Relative luciferase activity was calculated as the ratio of firefly to Renilla luciferase signal intensity. All values are mean ± SD of triplicate experiments. Data are from three independent experiments and presented as mean ± SD. ****p < 0.0001, ***p < 0.001, **p < 0.01, *p < 0.05.

qPCR analysis further verified significant mRNA upregulation in each overexpression group compared with controls (Figure 3A). Dual-luciferase assays following co-transfection of transcription factor expression plasmids with pGL4.10/exJSRV LTR revealed that FOXA1, FOXA2, FOXA3, and GATA3 significantly increased LTR promoter activity, with GATA3 producing the strongest enhancement (Figure 3B). Electrophoretic mobility shift assays (EMSA) confirmed that these four transcription factors specifically bound to the same upstream LTR region (−146/−132). The infrared fluorescence-labeled wild-type probe (T1-Wt-IR680) formed distinct protein–DNA complexes with nuclear extracts from OAR-L1 cells overexpressing FOXA1, FOXA2, FOXA3, or GATA3. The complexes were effectively competed by a 200-fold excess of unlabeled wild-type probe (T1-Wt) but not by the mutant competitor (T1-Mut) (Figure 3C). These findings indicate that the upstream LTR region contains binding sites for FOXA1, FOXA2, FOXA3, and GATA3, and that these transcription factors can specifically interact with the sequence *in vitro*.

### 3.4 GATA3 promotes the oncogenic potential of exJSRV LTR-env lentivirus

**Figure 4:**
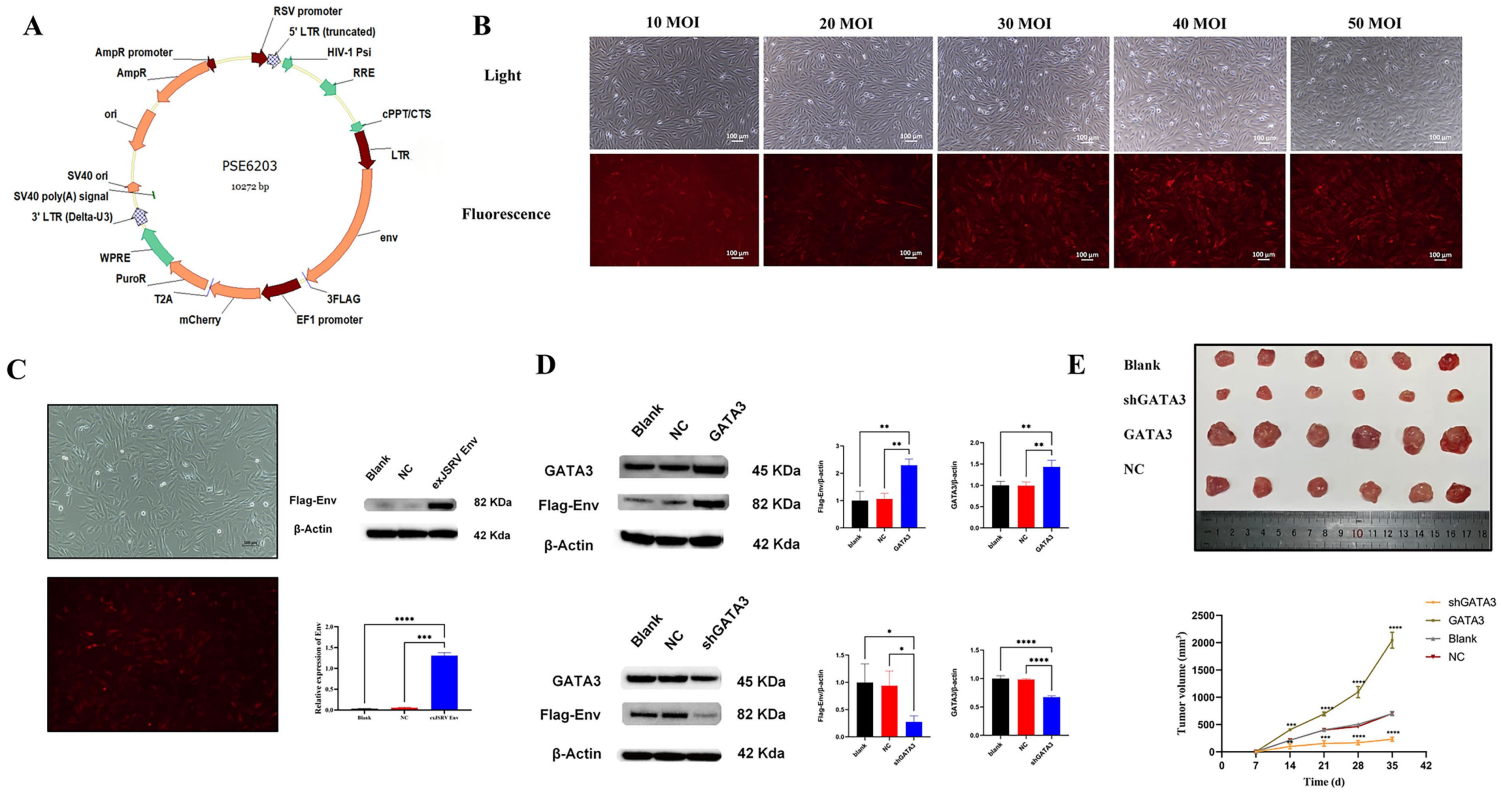
GATA3 may promote the oncogenic potential of exJSRV LTR-env lentivirus. **(A)** Schematic diagram of the exJSRV LTR-env lentiviral vector. **(B)** Fluorescence microscopy results of BEAS-2B cells transfected with exJSRV LTR-env lentivirus at different MOIs after 72 h. **(C)** Fluorescence expression and Western blot validation after puromycin selection. **(D)** Western blot analysis of exJSRV Env protein expression following GATA3 overexpression or knockdown. **(E)** Effect of GATA3 overexpression and knockdown on tumorigenic potential *in vivo* (*n* = 8). All values represent the mean ± SD. ****p < 0.0001, ***p < 0.001, **p < 0.01, *p < 0.05.

A schematic of the exJSRV LTR-env lentiviral vector is shown in Figure 4A. BEAS-2B cells were transduced at multiplicities of infection (MOI) ranging from 10 to 50. Based on red fluorescence intensity, transduction efficiency was most robust at MOIs of 40 and 50 (Figure 4B). To achieve high efficiency while maintaining cell viability, an MOI of 40 was selected for subsequent stable cell line generation. Transduced cells were then selected with 0.3 μg/mL puromycin for 7 d to establish a polyclonal population (Figure 4C). Western blot analysis confirmed Flag-tagged Env expression in the experimental group but not in the blank or negative controls (Figure 4C). To examine the regulatory role of GATA3, stable LTR-env cell lines were transfected with either GATA3 overexpression (p3xFLAG-CMV-14/GATA3) or knockdown (pGPU6/GFP/Neo-shGATA3) plasmids. Western blotting 48 h post-transfection revealed that Env protein levels were markedly elevated in the GATA3 overexpression group and reduced in the knockdown group compared with controls (Figure 4D). In the *in vivo* tumorigenicity assay, nude mice injected with LTR-env-overexpressing BEAS-2B cells developed visible tumors, confirming the oncogenic potential of the construct. Tumors from the GATA3 overexpression group were significantly larger than those from the control groups, whereas tumors in the GATA3 knockdown group were smaller (Figure 4E). These findings indicate that GATA3 enhances the tumorigenic potential of exJSRV LTR-env both *in vitro* and *in vivo*.

### 3.5 The exJSRV LTR demonstrated strong tropism towards the lungs and liver in mice

**Figure 5:**
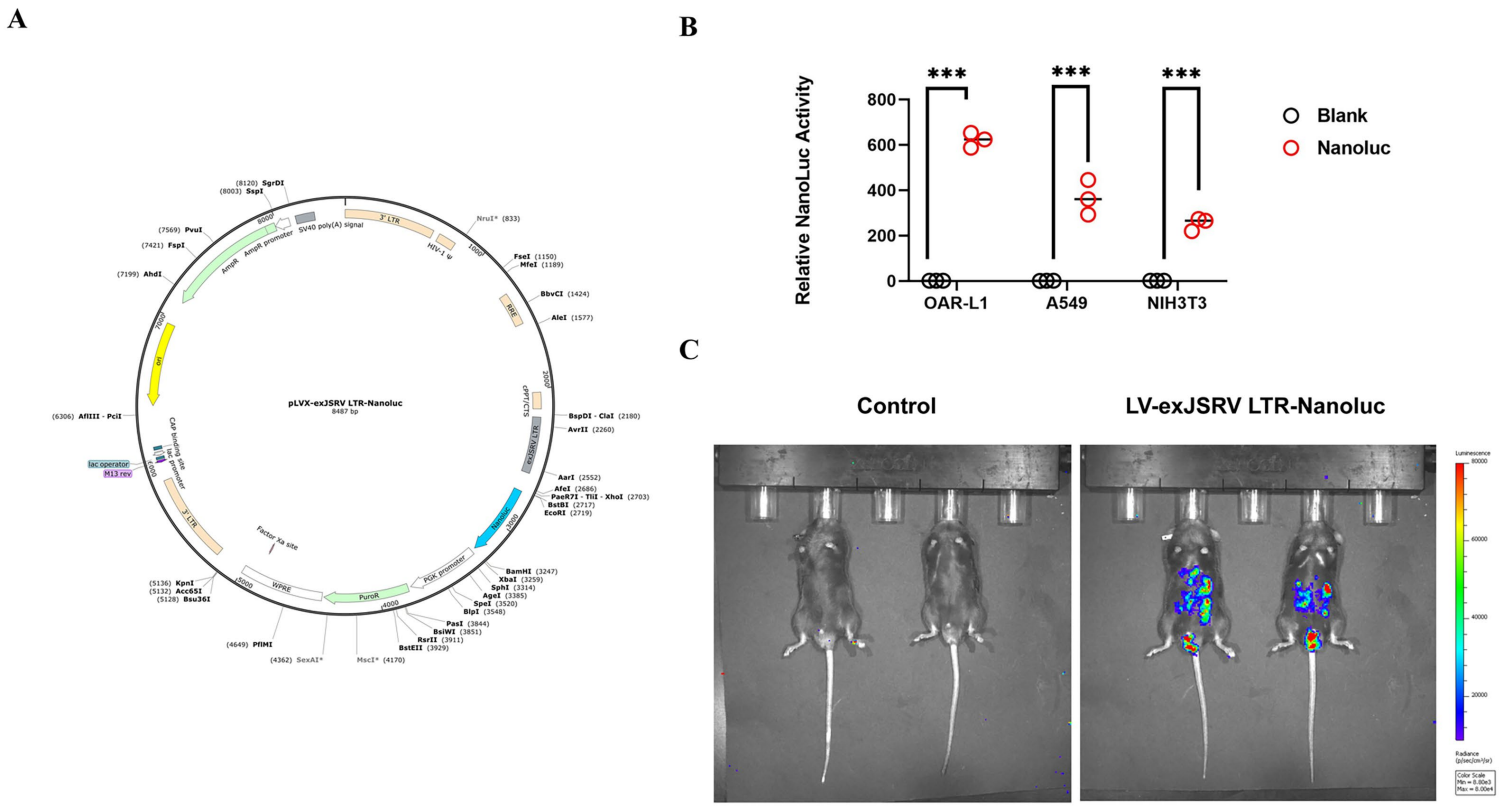
exJSRV LTR has tissue tropism for the liver and lungs of mice. **(A)** Structural schematic diagram of the recombinant plasmid pLVX-exJSRV LTR-nanoluc. **(B)** Determination of luciferase activity of the Nanoluc reporter gene in recombinant lentiviruses among three cell types. **(C)** Luminescence imaging of C57BL/6 mice was shown 21 d after tail vein injection of LV-exJSRV LTR-Nanoluc. All values represent the mean ± SD. ****p < 0.0001, ***p < 0.001, **p < 0.01, *p < 0.05.

To evaluate the tissue tropism of exJSRV LTR, the recombinant plasmid pLVX-exJSRV LTR-Nanoluc was constructed (Figure 5A). Lentiviral particles were produced by co-transfecting 293T cells with the expression plasmid and packaging vectors (pGag/Pol, pREV, and pVSV-G). After purification and concentration, the viral titer of LV-exJSRV LTR-Nanoluc was determined and used for infection assays. OAR-L1, A549, and NIH3T3 cells were infected at an MOI of 50 of LV-exJSRV LTR-Nanoluc. Seventy-two hours post-infection, Nanoluc luciferase activity was measured. In all three cell types, luciferase activity was significantly higher than in uninfected controls (*p* < 0.001), confirming efficient infection and expression of the Nanoluc reporter (Figure 5B). For *in vivo* analysis, C57BL/6 mice were injected via the tail vein with the LV-exJSRV LTR-Nanoluc. After 21 d, bioluminescence imaging revealed strong signals in the tail, lungs, and liver, while no signal was detected in the control group (Figure 5C). These results demonstrate that exJSRV LTR has potential tropism on the lungs and livers of mice.

## 4 Discussion

This study provides novel insights into the transcriptional regulation of the Jaagsiekte Sheep Retrovirus (exJSRV) long terminal repeat (LTR) and its role in the pathogenesis of Ovine Pulmonary Adenocarcinoma (OPA). Our findings reveal that host transcription factors, particularly GATA3 and FOXA2, together with the MEK/ERK signaling pathway, play a central role in controlling exJSRV LTR activity. These host factors not only regulate viral transcriptional efficiency but may also determine the tissue tropism and oncogenic potential of the virus in mammalian systems.

Through a combination of hTFtarget and AnimalTFDB analyses, we identified 53 potential transcription factors that could bind to the exJSRV LTR. KEGG enrichment analysis showed that these factors were mainly associated with the MAPK signaling pathway, a central regulator of cellular proliferation, differentiation, and survival (14–16). This finding aligns with previous studies reporting that several oncogenic viruses, such as HTLV-1 and Kaposi’s sarcoma-associated herpesvirus, exploit the MAPK/ERK cascade to enhance viral transcription and promote host cell transformation (17, 18). Our data further support this notion: activation of the MEK/ERK pathway by C16-PAF significantly increased exJSRV LTR promoter activity, whereas pharmacological inhibition by U0126 reduced it. This suggests that MEK/ERK signaling positively regulates exJSRV transcriptional activity, likely through phosphorylation of host transcription factors or chromatin modifiers that bind the LTR region. Such regulatory interactions may serve as a molecular link between viral gene expression and oncogenic signaling in infected epithelial cells.

Detailed analysis of the exJSRV LTR sequence identified a cluster of transcription factor binding motifs around the −147/−128 region, consistent with earlier observations that mutations in this region sharply decrease promoter activity (19, 20). We further examined the role of 18 candidate transcription factors predicted to bind this locus and discovered that FOXA1, FOXA2, FOXA3, and GATA3 were the most potent activators of LTR-driven expression. Among these, GATA3 exhibited the highest activation potential. These results are biologically meaningful, as both GATA and FOXA transcription factors are essential regulators of epithelial cell identity. They often function cooperatively to open compact chromatin and establish cell-type–specific enhancer landscapes (21). In the context of exJSRV infection, the co-enrichment of these factors at the LTR may create an open chromatin environment conducive to sustained viral transcription in pulmonary and hepatic tissues.

To confirm the biological significance of GATA3 in exJSRV regulation, we constructed an exJSRV LTR-env lentiviral vector and established a BEAS-2B cell line expressing the Env protein. Manipulating GATA3 levels directly influenced Env expression: GATA3 overexpression enhanced, whereas GATA3 knockdown significantly reduced, Env protein abundance. This clearly demonstrates that GATA3 acts as a transcriptional enhancer of exJSRV LTR activity. GATA3 is recognized as an oncogenic transcription factor in several cancers (22). In breast cancer, for instance, GATA3 maintains luminal differentiation and promotes chromatin remodeling through enhancer activation (23). Our study extends this oncogenic paradigm to a viral context, revealing that GATA3 enhances not only host oncogene transcription but also viral LTR activity, which may potentially promote virus-related tumorigenesis.

The *In vivo* tumorigenicity assay further supports this conclusion. BEAS-2B LTR-env xenografts exhibited faster tumor growth when GATA3 was overexpressed, whereas knockdown diminished tumor size. This suggests that GATA3 augments the oncogenic potential of exJSRV Env, possibly through combined transcriptional and signaling mechanisms. These findings are in line with reports that host transcription factors such as GATA3 can serve as critical co-factors in retroviral oncogenesis (24–26). Collectively, our results position GATA3 as a potential molecular node connecting host signaling, viral transcription, and tumor formation. Moreover, our study also revealed that the MEK/ERK signaling pathway represents another important regulator of exJSRV LTR activity. Previous studies have demonstrated that ERK-mediated phosphorylation can enhance the transactivation capacity of GATA family proteins (27), implying a potential synergistic interaction between MEK/ERK signaling and GATA3 in modulating exJSRV LTR activity. Such synergistic regulation has been documented in other retroviruses; for example, in HIV-1, MAPK activation amplifies Tat-mediated transcriptional elongation (28, 29). Thus, MEK/ERK–GATA3 crosstalk may constitute a conserved regulatory axis exploited by exJSRV to optimize viral replication and oncogenic efficiency in the host. However, this hypothesis warrants further experimental validation.

Although GATA3-mediated LTR activation explains much of the viral transcriptional pattern, it is likely not the sole regulatory mechanism. Other transcription factors identified in this study, such as FOXA2, may also participate in tissue-specific activation. FOXA2 is a pioneer factor critical for lung and liver development and has been shown to regulate surfactant protein expression and epithelial morphogenesis. It has previously been implicated in JSRV LTR regulation, where its binding to the U3 region enhances promoter activity in lung epithelial cells (19). This is consistent with our results demonstrating higher LTR activity in pulmonary and hepatic cells, suggesting that co-expression of FOXA2 and GATA3 in these tissues may underlie the observed organ tropism.

We also investigated the potential tissue tropism of exJSRV LTR using a recombinant LV-exJSRV LTR–Nanoluc reporter. *In vitro* assays revealed that OAR-L1, A549, and NIH3T3 cells all supported strong luciferase expression, confirming that exJSRV LTR is a robust promoter across multiple cell types. *In vivo*, bioluminescent imaging of C57BL/6 mice 21 d post-injection revealed pronounced signals in the lungs and liver, but not in other tissues. These results are consistent with the known lung tropism of JSRV LTR and with previous observations that AAV vectors containing the JSRV LTR display high expression in hepatic tissue (30, 31). The combined “lung + liver” tropism observed here suggests that exJSRV LTR activity is regulated by host transcriptional environments enriched in FOXA and GATA factors. Indeed, both GATA3 and FOXA2 are co-expressed in bronchiolar and hepatic epithelia, where they cooperatively maintain epithelial differentiation (32–35). This co-localization likely provides a favorable chromatin context for exJSRV transcription, facilitating persistent viral expression in these organs.

## 5 Conclusion

This study elucidates a regulatory framework in which host signaling pathways and transcription factors converge to modulate exJSRV transcription. We demonstrate that the MEK/ERK pathway and GATA3 directly regulate exJSRV LTR activity, delineate the LTR’s tissue tropism, and establish a pro-carcinogenic role for GATA3 in tumorigenesis. These findings deepen our molecular understanding of OPA pathogenesis and suggest novel therapeutic avenues. Specifically, we posit that pharmacological inhibition of MEK/ERK signaling or GATA3 function could suppress LTR-driven viral transcription and mitigate the oncogenic potential of exJSRV *in vivo*. Nevertheless, this study has limitations. We focused on a limited set of transcription factors due to resource constraints, and additional regulatory elements within the LTR remain to be characterized. In addition, the potential interaction between GATA3 and the MEK/ERK signaling pathway is worthy of further research and confirmation.

## Data Availability

The data generated by this study can be sourced from articles and Supplementary materials.

## Footnotes

1
https://guolab.wchscu.cn/hTFtarget/#!/tf

2
https://guolab.wchscu.cn/AnimalTFDB4/#/TF_Predict

## Supplementary material

SUPPLEMENTARY TABLE S1 exJSRV LTR Sequence.

SUPPLEMENTARY TABLE S2 Relevant primer and probe sequences.

SUPPLEMENTARY TABLE S3 Transcription factor prediction.
