# Supplementary material for: Investigation of exJSRV LTR promoter activity based on transcription factor regulatory networks
Source: Front Vet Sci. 2026 Jan 9;12:1727983. doi: 10.3389/fvets.2025.1727983 (PMC12827560; doi:10.3389/fvets.2025.1727983)
Supplement: SUPPLEMENTARY TABLE S1 — exJSRV LTR Sequence. [file Table_1.DOCX]

GATGCGGGGGACGACCCGTGAAGGGTTAAGTCCTGGGAGCTCTTTGGCAGAAGCCAAAGCCTAGGACAAGTACCTAAGCTCCCTGTCCCGCCACCCTCAAGAATTTTTAAAAGCTCTTAAGGCTCGGATGTTTGCTTTTGGCACTGCTTCACAGAAATACCAGGAAATCTGATTATATAAGAATCCGGTGATTGTGTAAGAATCCGGTGGGTGTAGCTTATAATGAATAAACAAGTTATGTTACTTTATAAATATAGCATTGTAATAAAGCAGAGTATCAGCCGTTTTGGTCTGATCCTCTCAACCCCATCTTTTGTCTCTCTCTCGTTTCTTAGCGGGGACGCTCCGTTCTCTCCCTGTGCAGGTGCGACTCTTGCTTGTGCTGGCCGCGGCAGG
