# Supplementary material for: Investigation of exJSRV LTR promoter activity based on transcription factor regulatory networks
Source: Front Vet Sci. 2026 Jan 9;12:1727983. doi: 10.3389/fvets.2025.1727983 (PMC12827560; doi:10.3389/fvets.2025.1727983)
Supplement: SUPPLEMENTARY TABLE S2 — Relevant primer and probe sequences. [file Table_2.DOCX]

The amplification primer sequence

| Name | Sequence (5′→3′) | Length(bp) |
| --- | --- | --- |
| T1 | F:TTAAGGCTGGCACTGCTTCACAGAAATACCAGG  R:AAGCAGTGCCAGCCTTAAGAGCTTTTAAAAATTCTTG | 4257 |
| T2 | F:TAAGAATCCGGTGATCCGGTGGGTGTAGCTTATAATG  R:CGGATCACCGGATTCTTATATAATCAGATTTCC | 4255 |

The amplification primer sequence of LTR

|  | Name | Sequence (5′→3′) | Enzyme cleavage site | Length(bp) |
| --- | --- | --- | --- | --- |
| The first segment | exLTR-F1 | CGGGGTACCGATGCGGGGGACGACCCGTGAA | *Kpn* I | 209 |
|  | exLTR-R1 | CACCGGATTCTTACACAATCACCGG | - |  |
| Thesecondsegment | exLTR-F2 | CCAGGAAATCTGATTATATAAGAATCCGG | - | 237 |
|  | exLTR-R2 | CCCAAGCTTCCTGCCGCGGCCAGCACAA | *Hin*d Ⅲ |  |

Specific primer sequence

| Gene | Sequence(5′→3′) | Enzyme | Length(bp) |
| --- | --- | --- | --- |
| FOXA1 | F:CCCAAGCTTatgttagggactgtgaagatgg | *Hin*d Ⅲ | 1404 |
|  | R:CTAGTCTAGAagaagtgtttagaacgggtctg | *Xba* Ⅰ |  |
| FOXA2 | F:CCCAAGCTTatgcactcggcttccagtatg | *Hind* Ⅲ | 1401 |
|  | R: R:CGCGGATCCcgaggagttcatgatgg | *BamH* I |  |
| FOXA3 | F:CCCAAGCTTatgctgggctcggtgaagatg | *Hind* Ⅲ | 1053 |
|  | R:CTAGTCTAGAggatgcgttaagcagagagcg | *Xba* Ⅰ |  |
| GATA3 | F:CCCAAGCTTatggaggtgacggcggac | *Hind* Ⅲ | 1329 |
|  | R:CTAGTCTAGAgcccatggcggtgaccata | *Xba* Ⅰ |  |
| AR | F:CGGGGTACCatggaagtgcagttagggctg | *Kpn* I | 2670 |
|  | R:CGCGGATCCctgagtgtgaaaatagataggcttg | *BamH* I |  |
| ARNT | F:CCCAAGCTTatggcggcgactactgctaac | *Hind* Ⅲ | 2370 |
|  | R:CTAGTCTAGAttctgaaaaggagggaaacatagtt | *Xba* Ⅰ |  |
| CTCF | F:CCCAAGCTTatggaaggggaaatggaaggtg | *Hind* Ⅲ | 2193 |
|  | R:CTAGTCTAGAccggtccatcatgctgagga | *Xba* Ⅰ |  |
| ERG | F:CCCAAGCTTatgttgccttcattttgtggaacc | *Hind* Ⅲ | 1491 |
|  | R:CTAGTCTAGAgtagtaagtgcccagatgcga | *Xba* Ⅰ |  |
| FOXP1 | F:CCCAAGCTTatgatgcaagaatctgggactg | *Hind* Ⅲ | 2025 |
|  | R:CTAGTCTAGActccatgtcctcatttacaggt | *Xba* Ⅰ |  |
| FOXJ2 | F:CGGGGTACCatggcttctgacctagagagc | *KPn* Ⅰ | 1683 |
|  | R:CGCGGATCCggtaatcaagtcccaatcgaag | *BamH* Ⅰ |  |
| FOXJ3 | F:AAGGAAAAAAGCGGCCGCatgggtttgtatggacag | *Not* Ⅰ | 1875 |
|  | R:CGCGGATCCcacaattgaatcccaatcaaagtcat | *BamH* Ⅰ |  |
| HDAC3 | F:CCCAAGCTTatggccaagactgtggcctat | *Hind* Ⅲ | 1284 |
|  | R:CGCGGATCCaatctccacgtcgctttccttg | *BamH* I |  |
| NFIA | F:AAGGAAAAAAGCGGCCGCatggatgaatttcatcctttcatcg | *Not* Ⅰ | 1503 |
|  | R:CTAGTCTAGAtcccaggtaccaggactgtg | *Xba* Ⅰ |  |
| PIAS1 | F:CCCAAGCTTatgagatgttgggtggaacaag | *Hind* Ⅲ | 1962 |
|  | R:CGCGGATCCgtccaatgaaataatgtctggtatg | *BamH* I |  |
| SP1 | F:CCCAAGCTTatgagcgaccaagatcactcc | *Hind* Ⅲ | 2358 |
|  | R:CTAGTCTAGAgaagccattgccactgatattg | *Xba* Ⅰ |  |
| TCF12 | F:AAGGAAAAAAGCGGCCGCatgaagcagttaaatagcaaagcaag | *Not* I | 1944 |
|  | R:CGCGGATCCcatatgacccatagggttggc | *BamH* I |  |
| RARA | F:CCCAAGCTTatgtacgagagtgtggacgtggggg | *Hind* Ⅲ | 1377 |
|  | R:CTAGTCTAGAcggggagtgggtggccgggctgctt | *Xba* Ⅰ |  |
| RXRA | F:CTAGTCTAGAgtctttggtttctaagtttaaaggg | *Hind Ⅲ* | 1317 |
|  | R: CTAGTCTAGAagtcatctggtgcggggc | *Xba Ⅰ* |  |

Near infrared fluorescent probe sequence

| Probe | Sequence |
| --- | --- |
| T1-Wt-IR680  T1-Wt | IR680-5’CTTAAGGCTCGGATGTTTGCTTTTGGCACT  5’CTTAAGGCTCGGATGTTTGCTTTTGGCACT |
| T1-Mut | 5’CTTAAGGCTGAAGCCCGCCCCTTTGGCACT |

Fluorescent quantitative PCR primer sequence

| 引物名称 | 引物序列(5′→3′) | 扩增长度/bp |
| --- | --- | --- |
| FOXA1 | F：AAGCCGCCCTACTCGTACATCTC  R：GCTGCTGGTTCTGCCGGTAATAG | 130 |
| FOXA2 | F:ATGCACTCGGCTTCCAGTAT  R:CATGTTGCTCACCGAGGAGT | 111 |
| FOXA3 | F：GGAGGCGGGCGAGGTCTATTC  R：AGCGGGTTCAGGGTCATGTAGG | 81 |
| GATA3 | F：CACCACCTACCCGCCCTACG  R: CCTGTGCTGGATCTCGCCTTG | 123 |
| AR | F：TGAGGAGCCAGCCCAGAAGC  R：CTGCGAAGGAGTCAGGTTGGTTG | 140 |
| ARNT | F：ATTTGCCAGGTCGGATGATGAGC  R：CCGTCGTCGCCGTTCAATCTC | 88 |
| CTCF | F：TTGGTGCGGCATCGTCGTTAC  R：CCCAGTGTGAGAGCGAATGTGAC | 117 |
| ERG | F：GGGAAGGAGCTGTGCAAGATGAC  R：TGGAGGTGCGACAGGAGGATG | 86 |
| FOXP1 | F：AGCAGCAGCAGGCACTTCAAG  R：AAGGGCTGGCTGTTTGTCATTCC | 110 |
| FOXJ2 | F：CTGGTGGGAGCCGCAAATGTC  R：AGGTGATGAGGGTGGCGTAGC | 124 |
| FOXJ3 | F：GCACTCAGCACTCCAGGAACAAC  R：GGAAGGCTTGGGAAGGCATCATG | 82 |
| HDAC3 | F：TGGACCAGATCCGCCAGACAATC  R：TCCTCCGCATCAGCCTCATCAG | 127 |
| NFIA | F：CATGAAGTGGAGCCAGGAATGCC  R：GGAGGAGGTCTGTGAAGGAGAGG | 93 |
| PIAS1 | F：CTCTGCCAACCACCAACGGAAG  R：TGCTGCTCCTATTTGCGACAGTG | 105 |
| SP1 | F：ACAACTTTCCCAGGGTGCCAATG  R：CTGCTGCCACTCTGTTCCTTCG | 87 |
| RARA | F：GCTTCACCACCCTCACCATTGC  R：AGCCCGTCCGAGAAGGTCATC | 127 |
| RXRA | F：GAGCAGCAGCGAGGACATCAAG  R：AGGACGCCATGTTCCCTGAGG | 86 |
| TCF12 | F：TCAGGCCGTGGCAGTCATCC  R：GCAGTGTGGTTGGTGGCTCTG | 134 |
