# Supplementary material for: Investigation of exJSRV LTR promoter activity based on transcription factor regulatory networks
Source: Front Vet Sci. 2026 Jan 9;12:1727983. doi: 10.3389/fvets.2025.1727983 (PMC12827560; doi:10.3389/fvets.2025.1727983)

Figure 2B

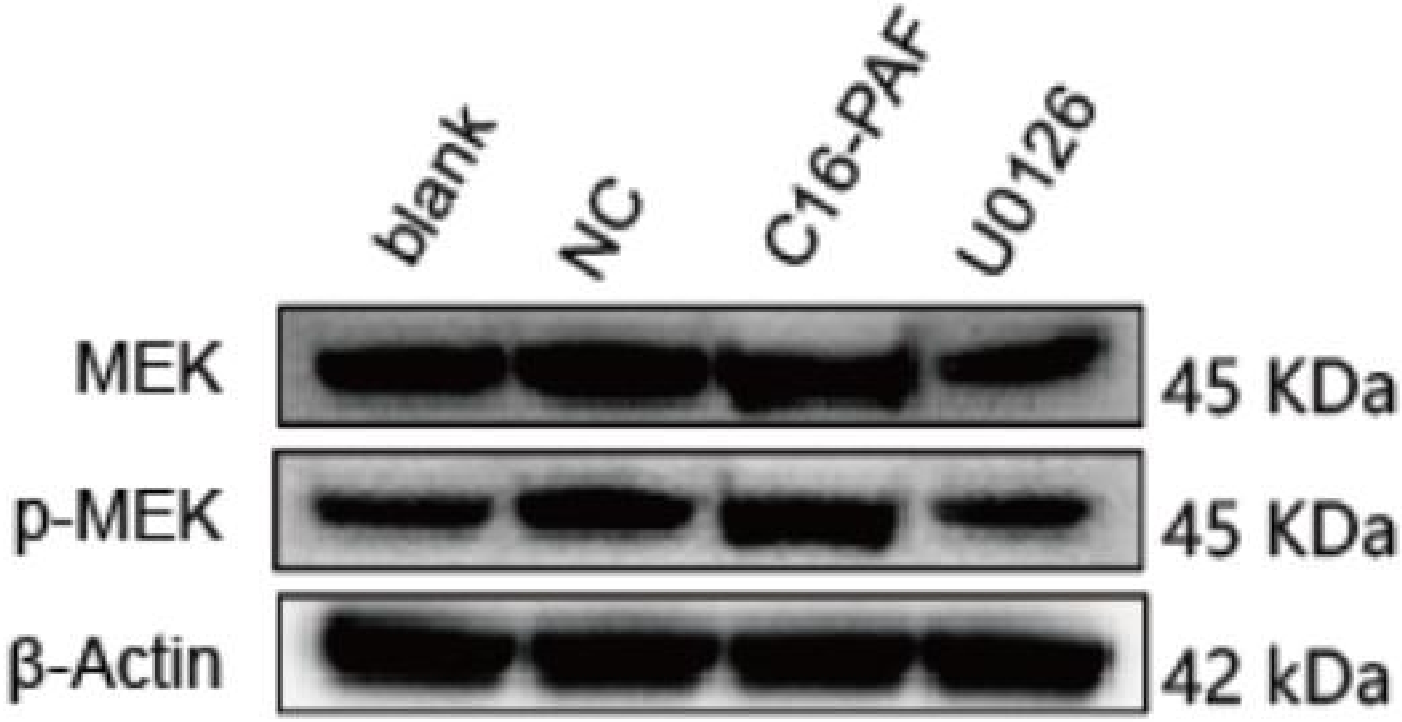

Figure 2B

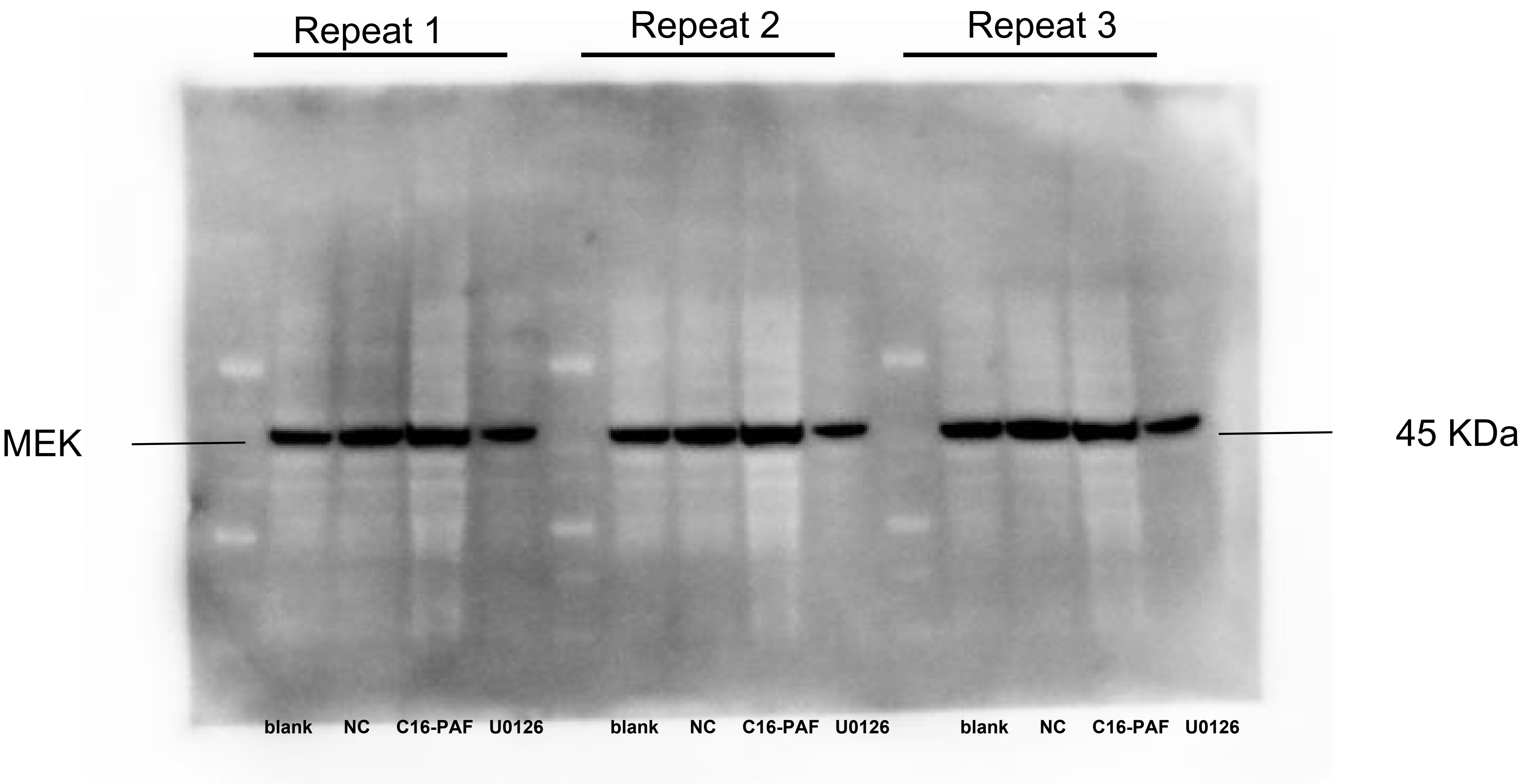
Repeat 1
Repeat 2
Repeat 3
MEK
45 KDa
blank
NC
C16-PAF
U0126
blank
NC
C16-PAF
U0126
blank
NC
C16-PAF
U0126

Figure 2B

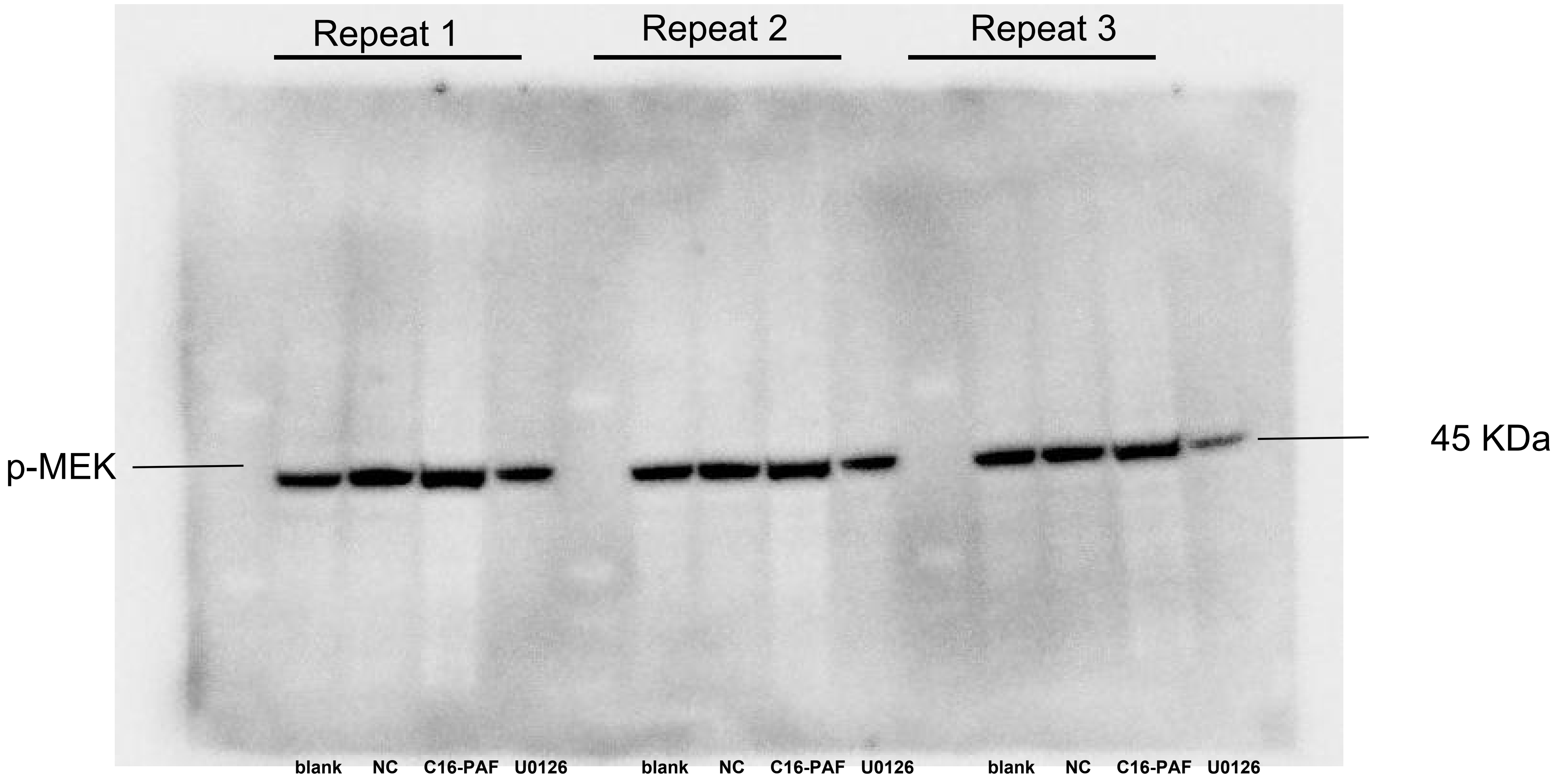
Repeat 1
Repeat 2
Repeat 3
p-MEK
45 KDa
blank
NC
C16-PAF
U0126
blank
NC
C16-PAF
U0126
blank
NC
C16-PAF
U0126

Figure 2B

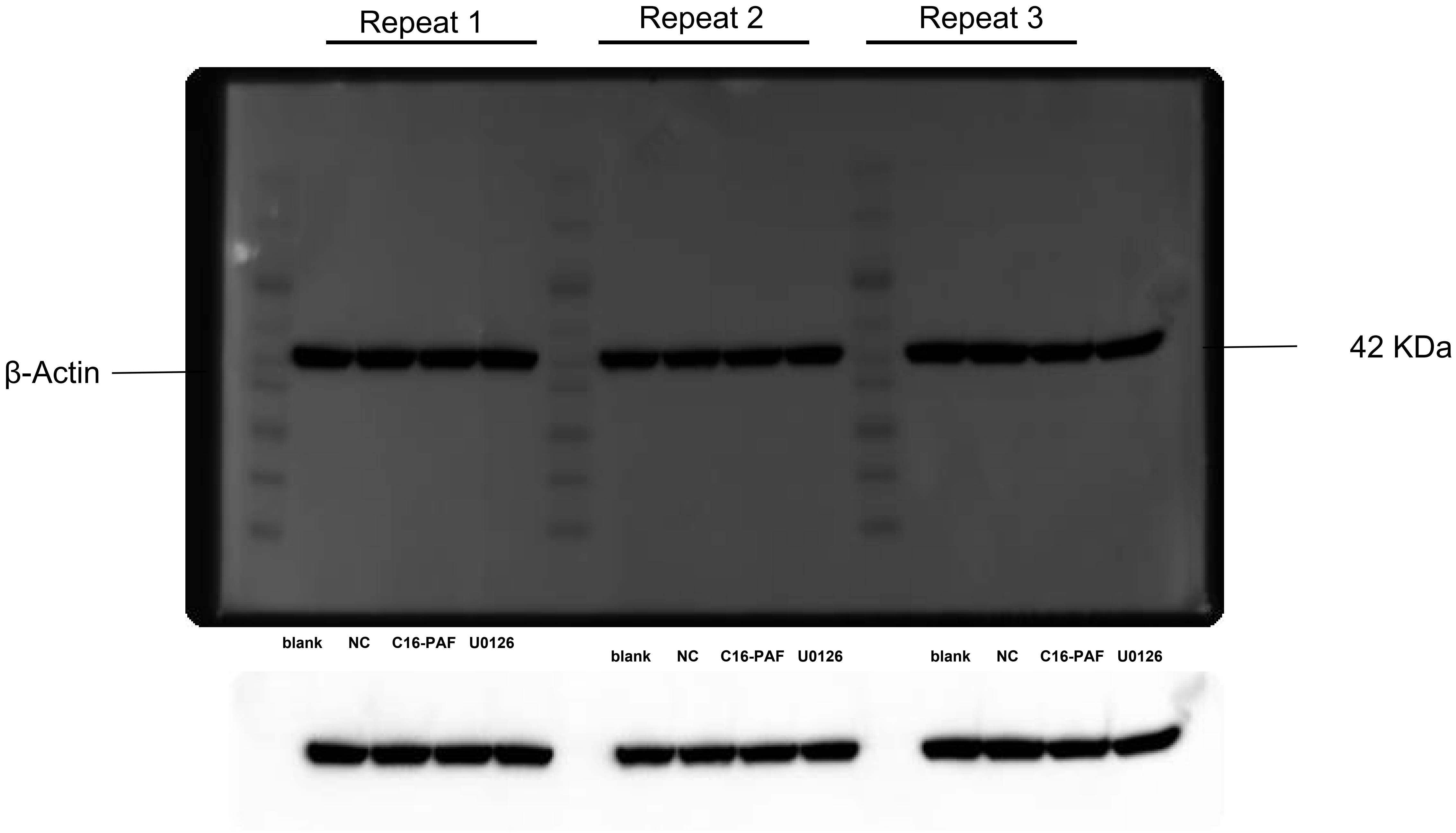

Repeat 1
Repeat 2
Repeat 3
β-Actin
42 KDa
blank
NC
C16-PAF
U0126
blank
NC
C16-PAF
U0126
blank
NC
C16-PAF
U0126

Figure 2C

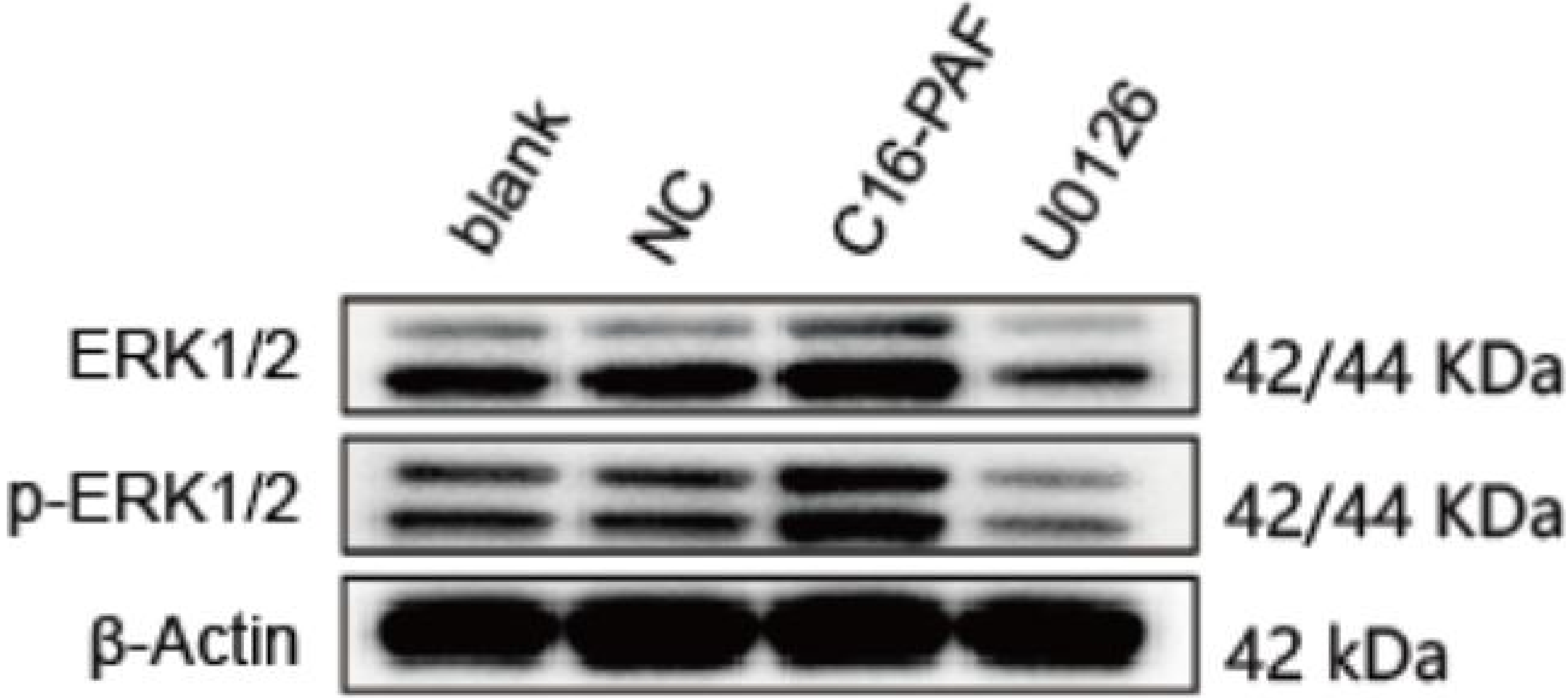

blank
NC
C16-PAF
U0126
ERK1/2
42/44 KDa
p-ERK1/2
42/44 KDa
β-Actin
42 kDa

Figure 2C

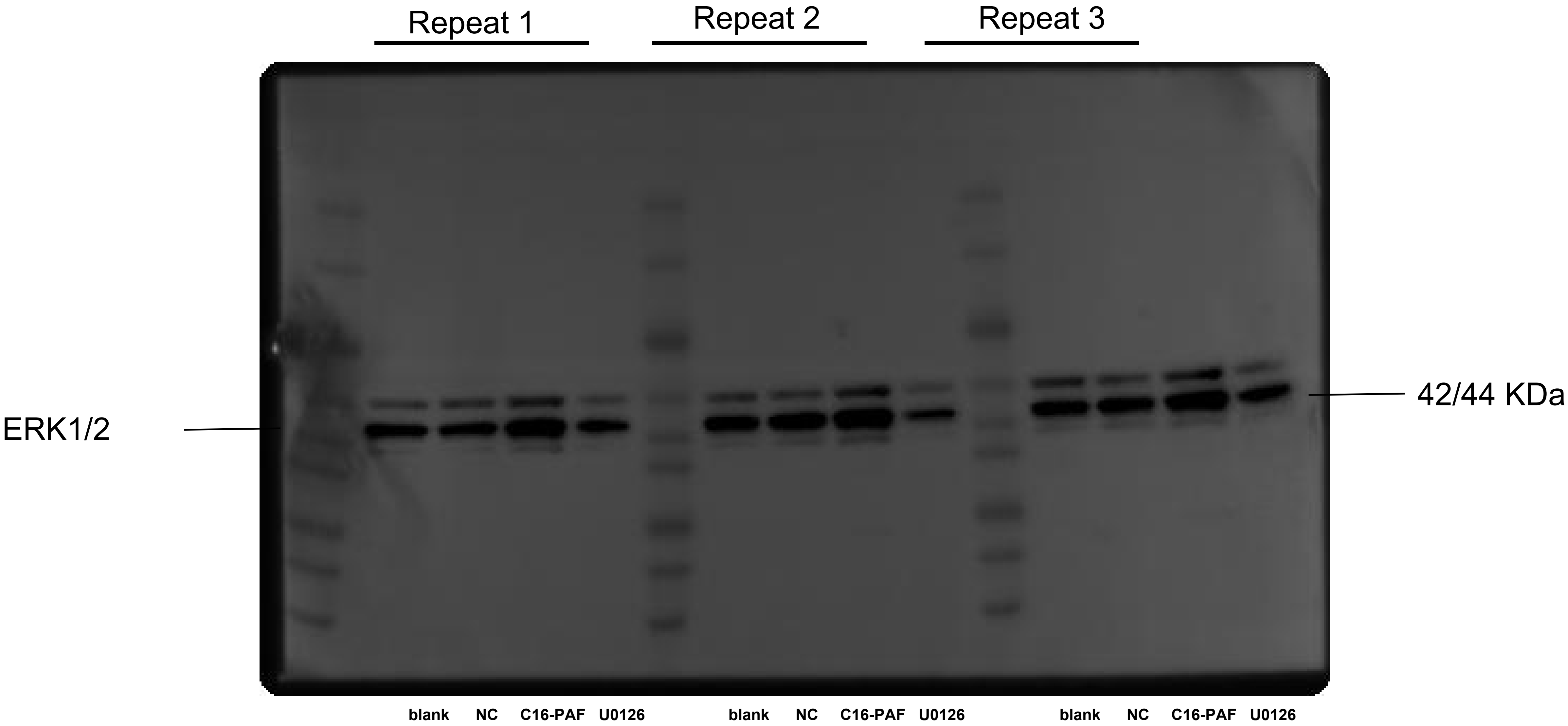

Repeat 1
Repeat 2
Repeat 3
ERK1/2
42/44 KDa
blank
NC
C16-PAF
U0126
blank
NC
C16-PAF
U0126
blank
NC
C16-PAF
U0126

Figure 2C

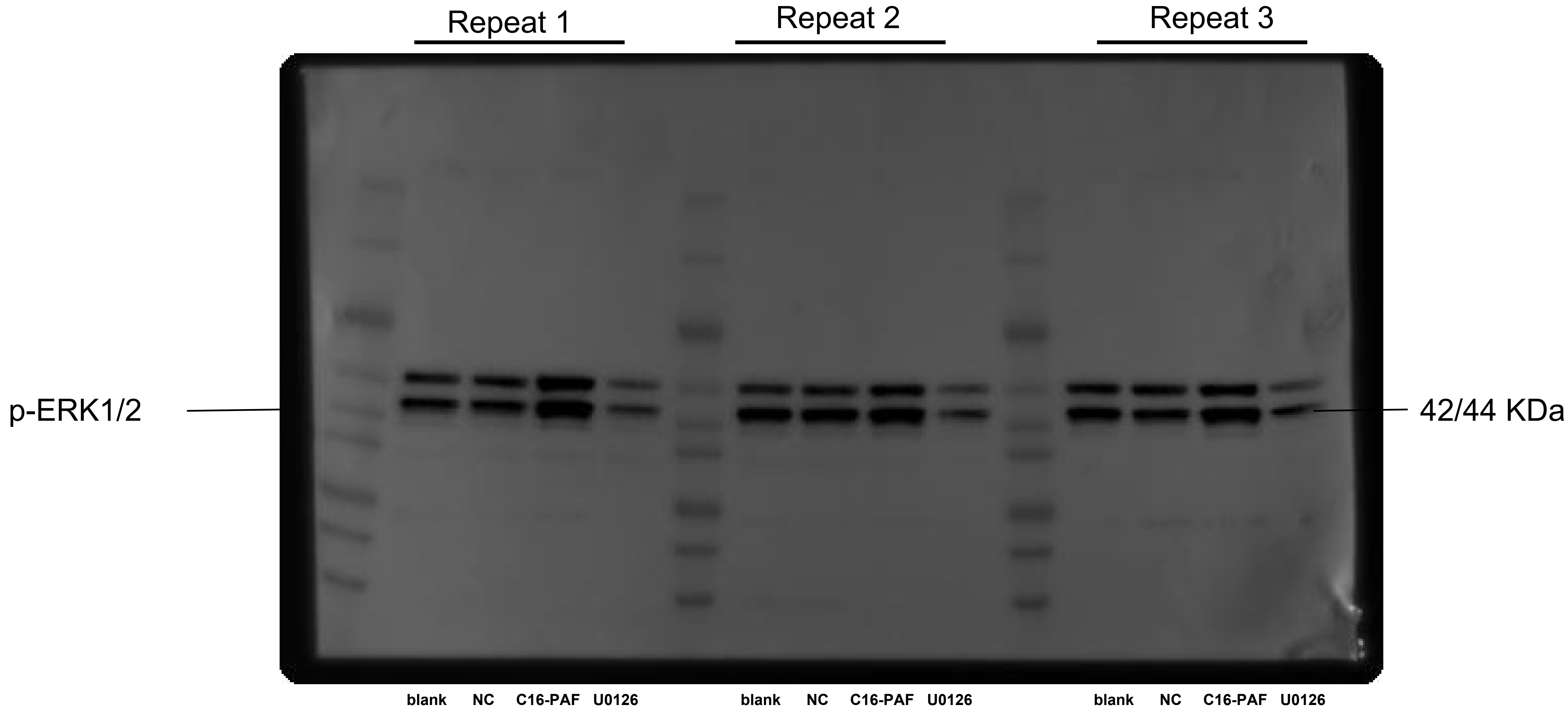
Repeat 1
Repeat 2
Repeat 3
p-ERK1/2
42/44 KDa
blank
NC
C16-PAF
U0126
blank
NC
C16-PAF
U0126
blank
NC
C16-PAF
U0126

Figure 2C

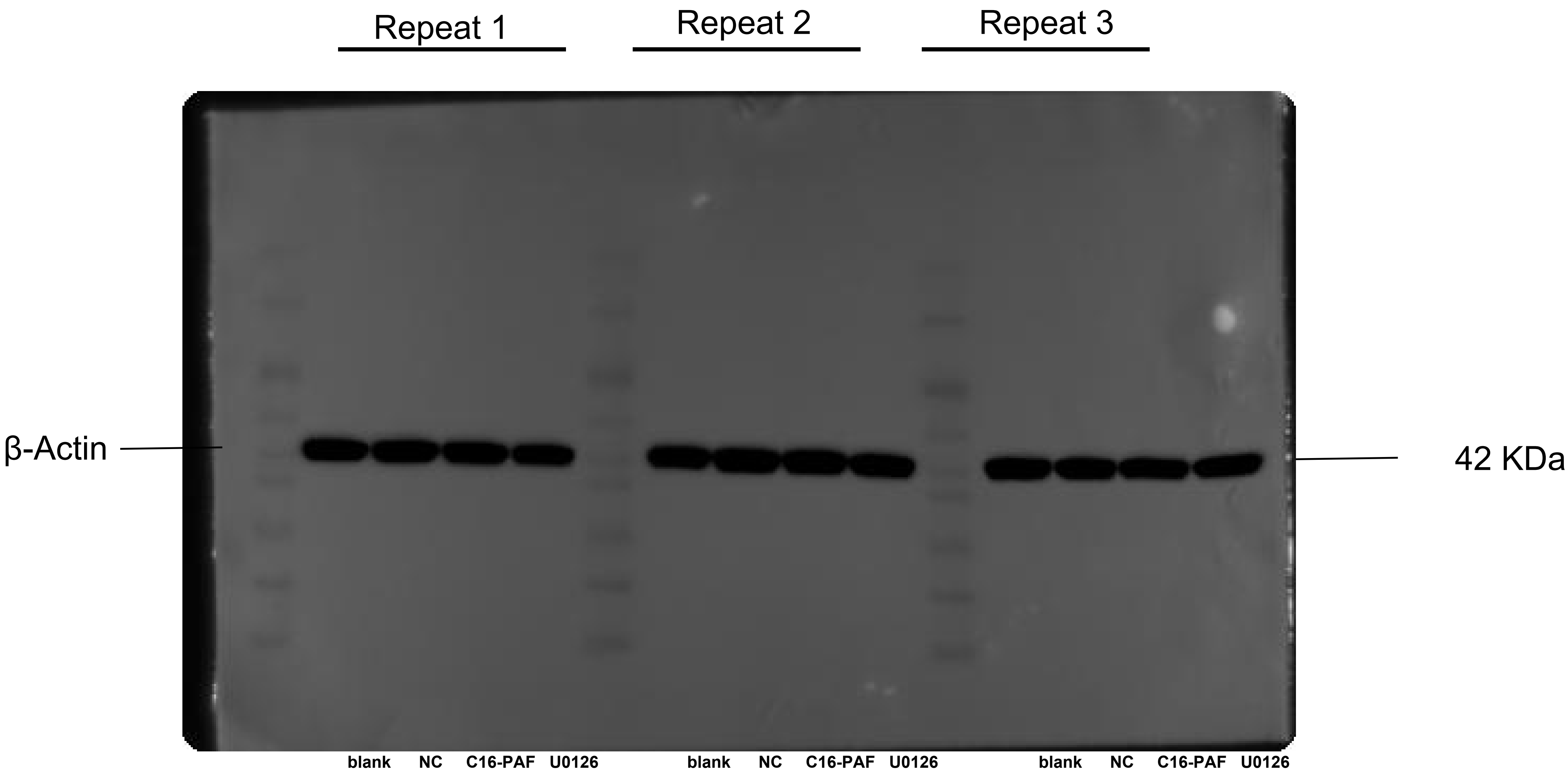
Repeat 1
Repeat 2
Repeat 3
β-Actin
42 KDa
blank
NC
C16-PAF
U0126
blank
NC
C16-PAF
U0126
blank
NC
C16-PAF
U0126

Figure 4C

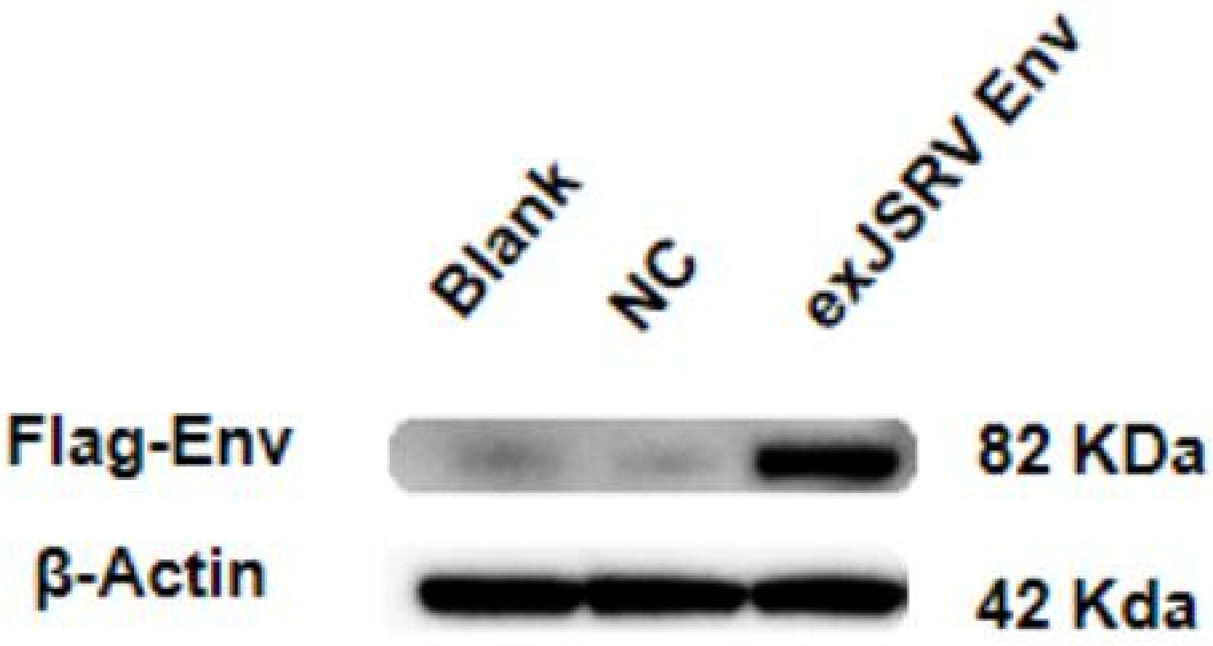

Figure 4C

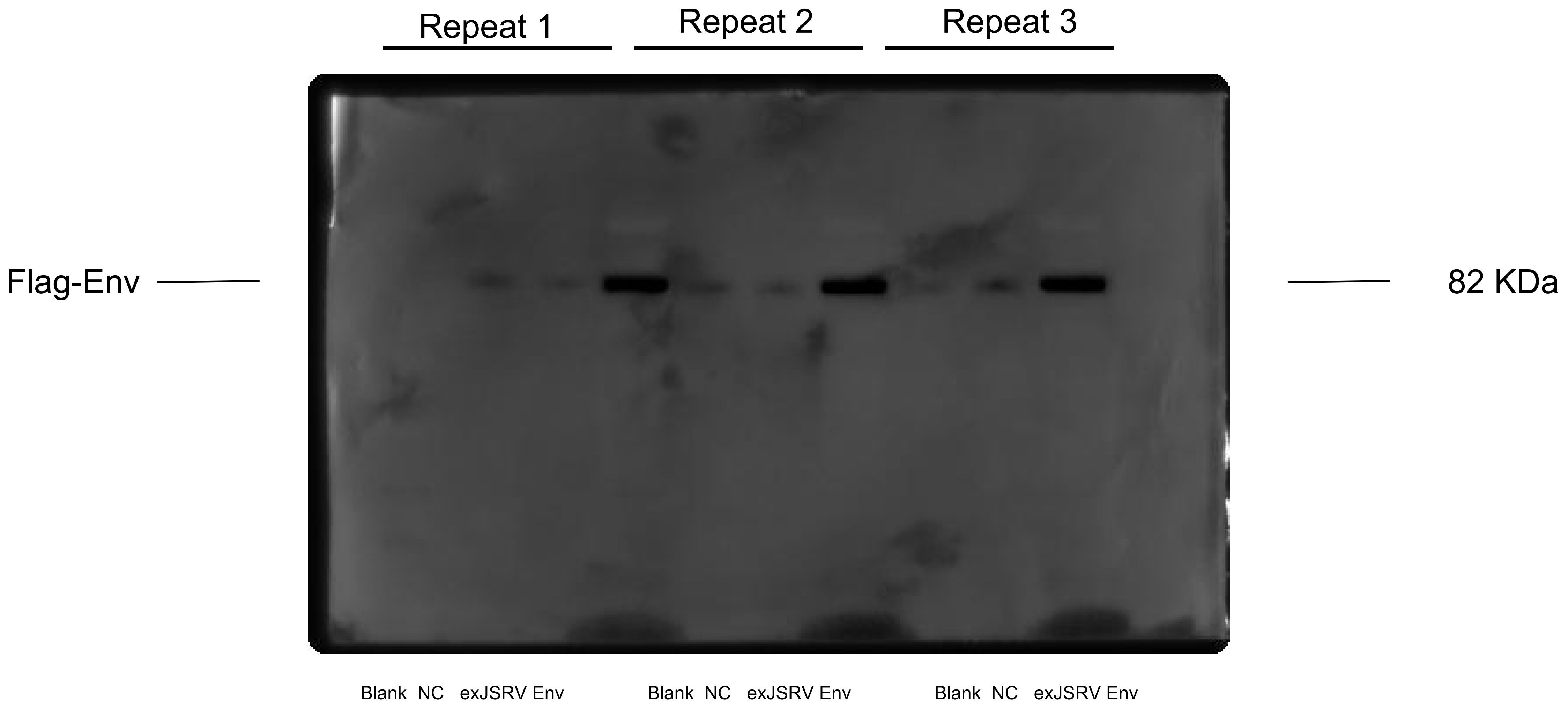
Repeat 1
Repeat 2
Repeat 3
Flag-Env
82 KDa
Blank NC exJSRV Env
Blank NC exJSRV Env
Blank NC exJSRV Env

Figure 4C

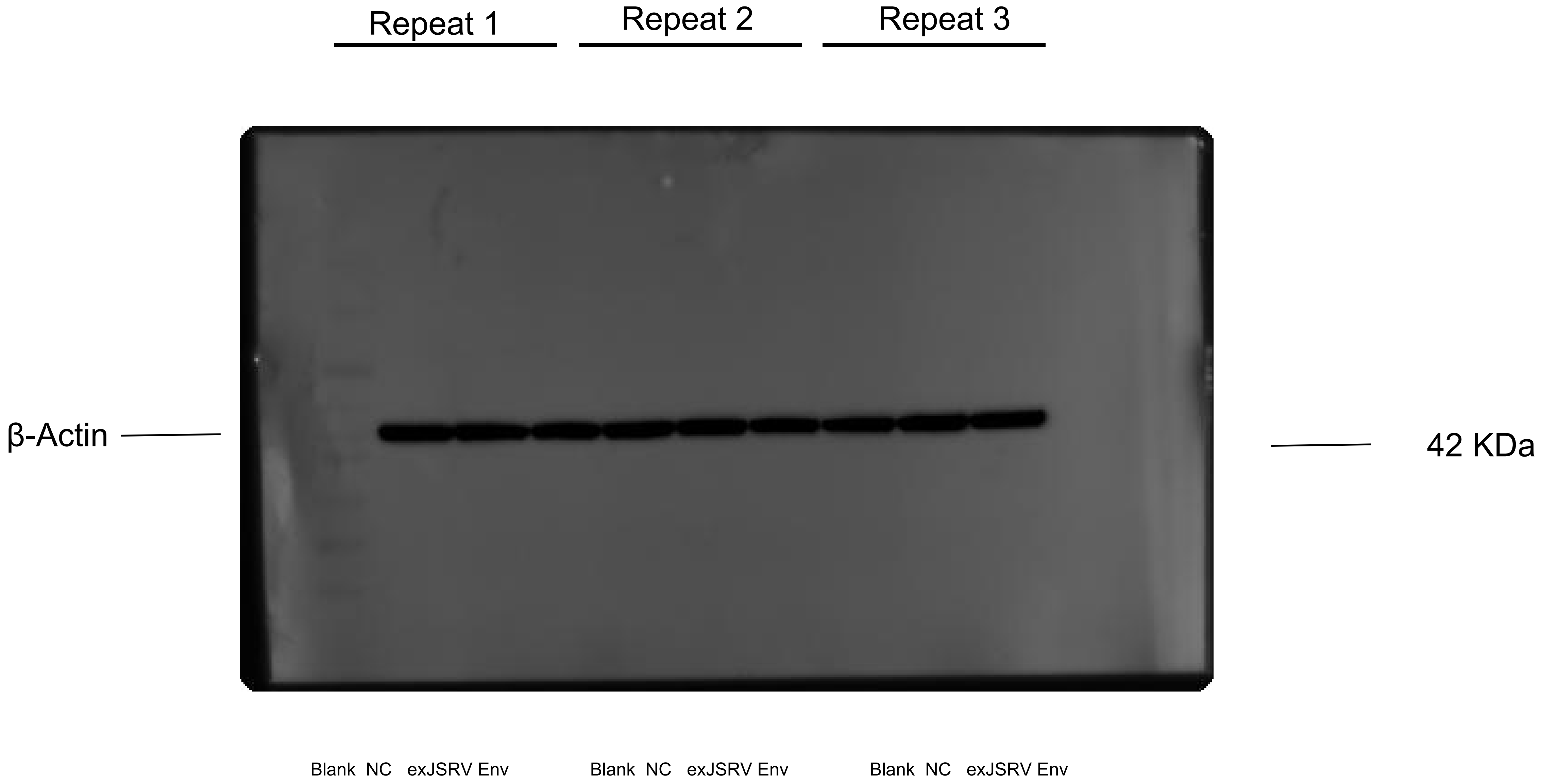

Repeat 1
Repeat 2
Repeat 3
β-Actin
42 KDa
Blank NC exJSRV Env
Blank NC exJSRV Env
Blank NC exJSRV Env

Figure 4D

Blank NC GATA3

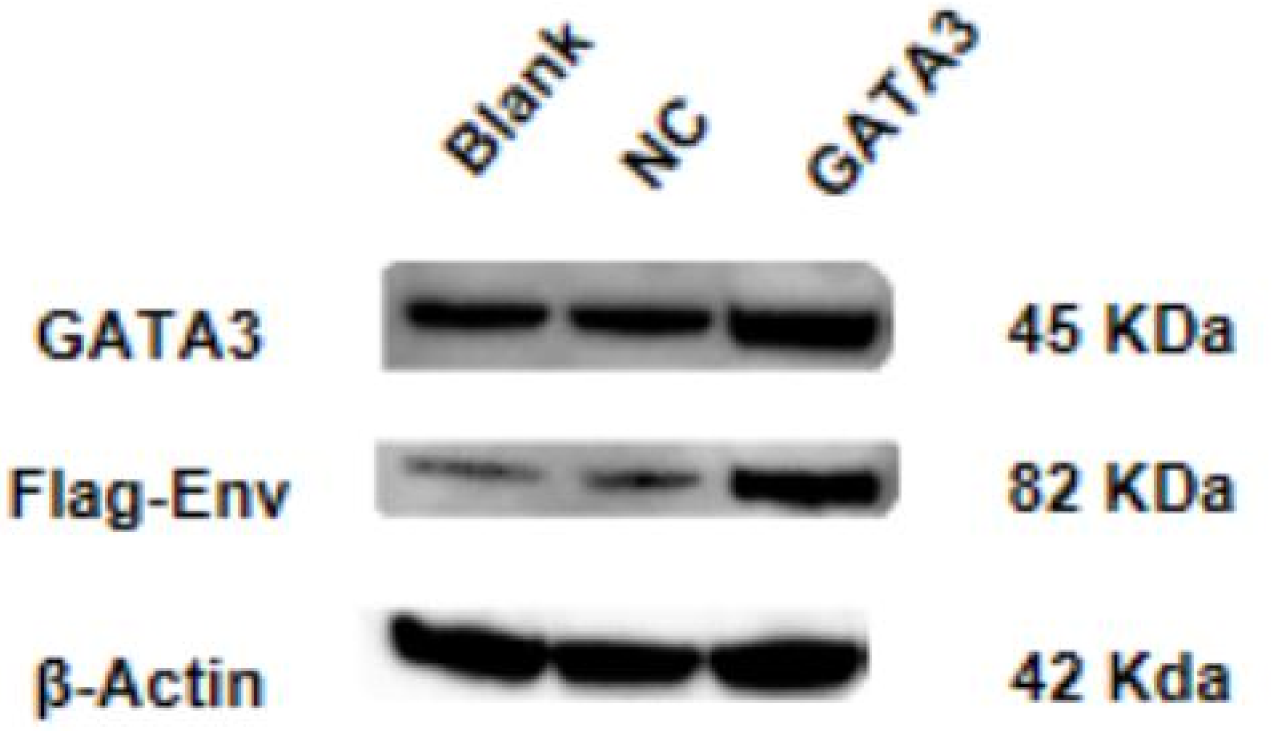

Figure 4D

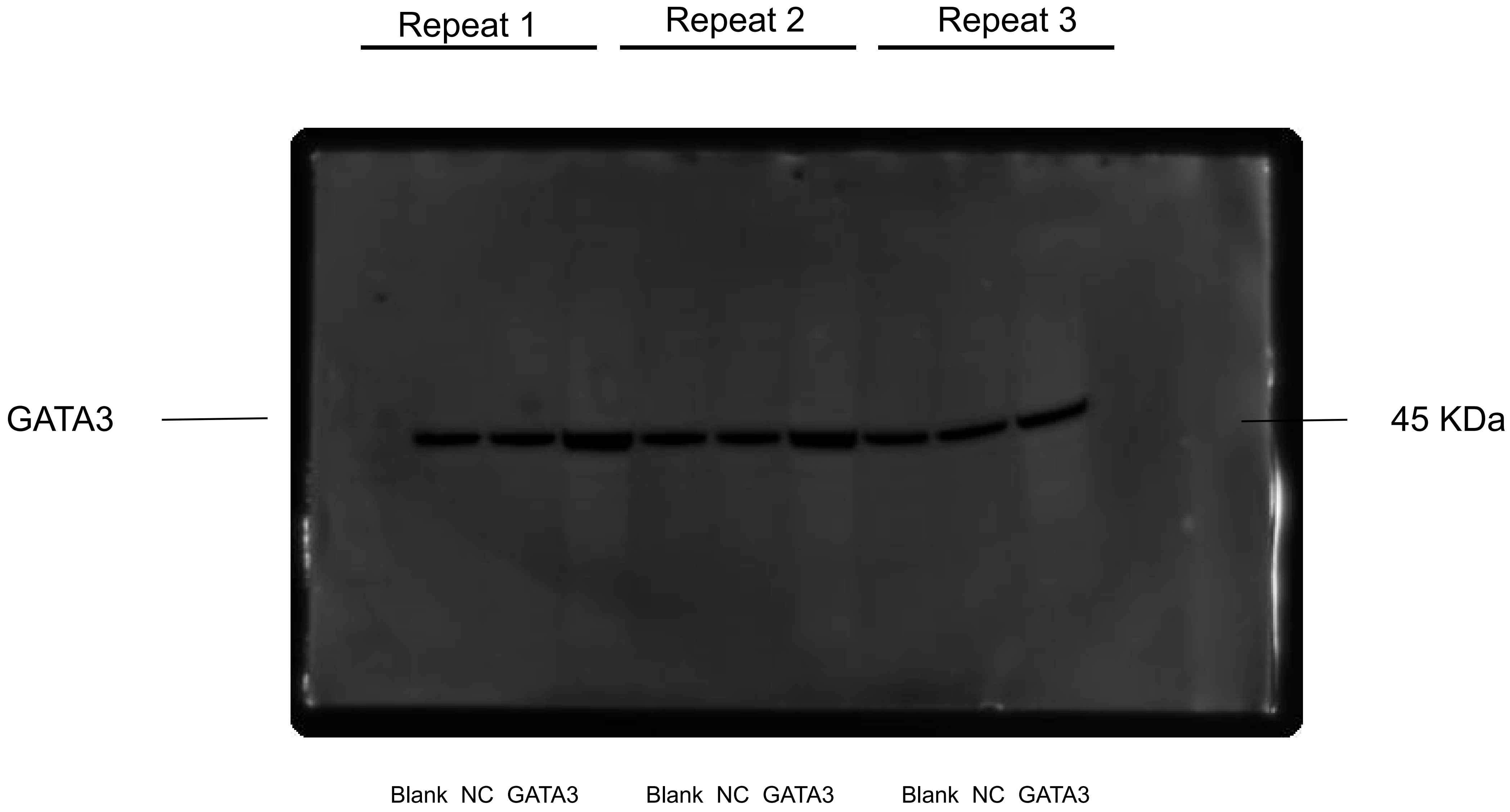
Repeat 1
Repeat 2
Repeat 3
GATA3
45 KDa
Blank NC GATA3
Blank NC GATA3
Blank NC GATA3

Figure 4D

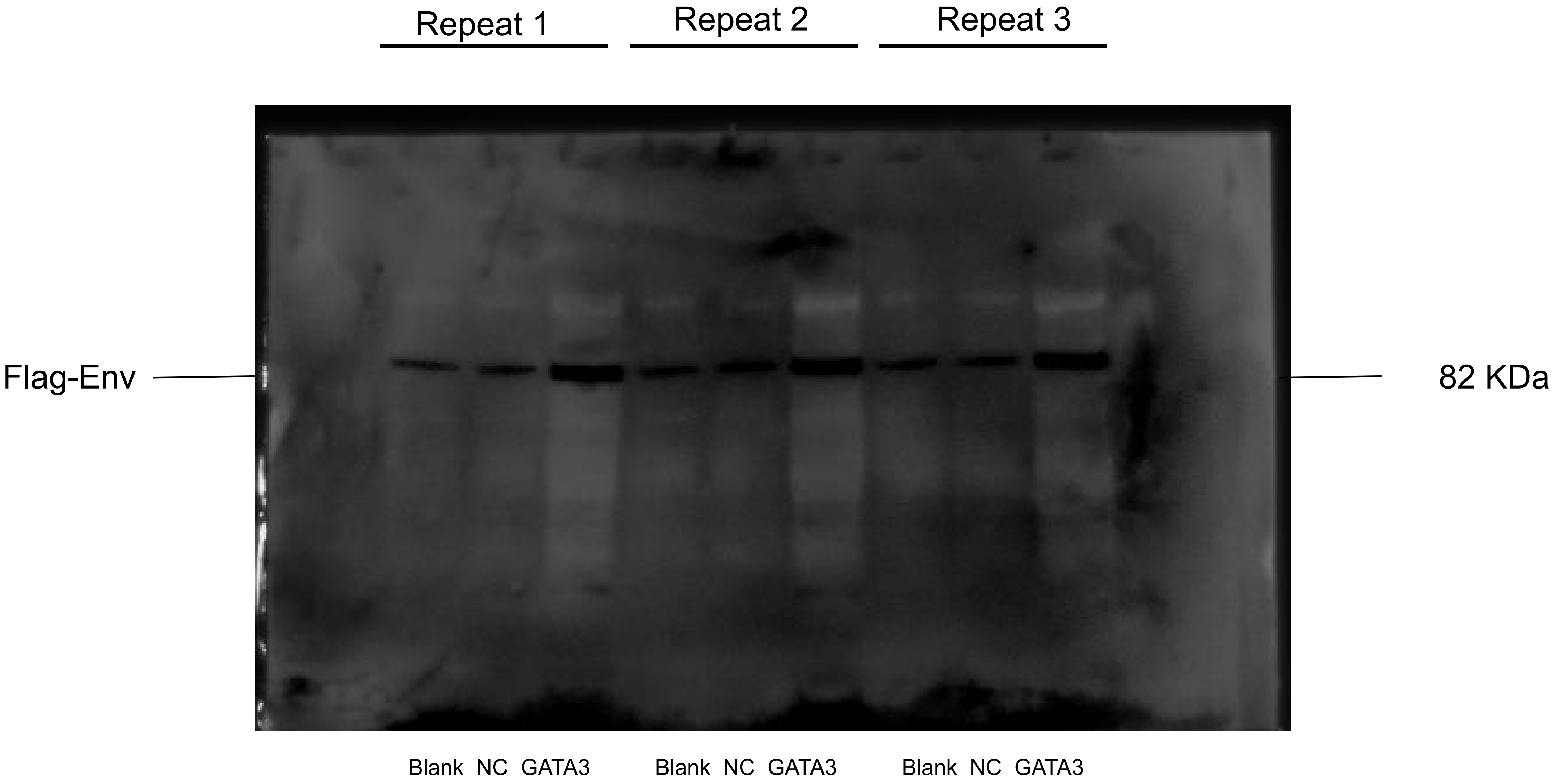

Repeat 1
Repeat 2
Repeat 3
Flag-Env
82 KDa
Blank NC GATA3
Blank NC GATA3
Blank NC GATA3

Figure 4D

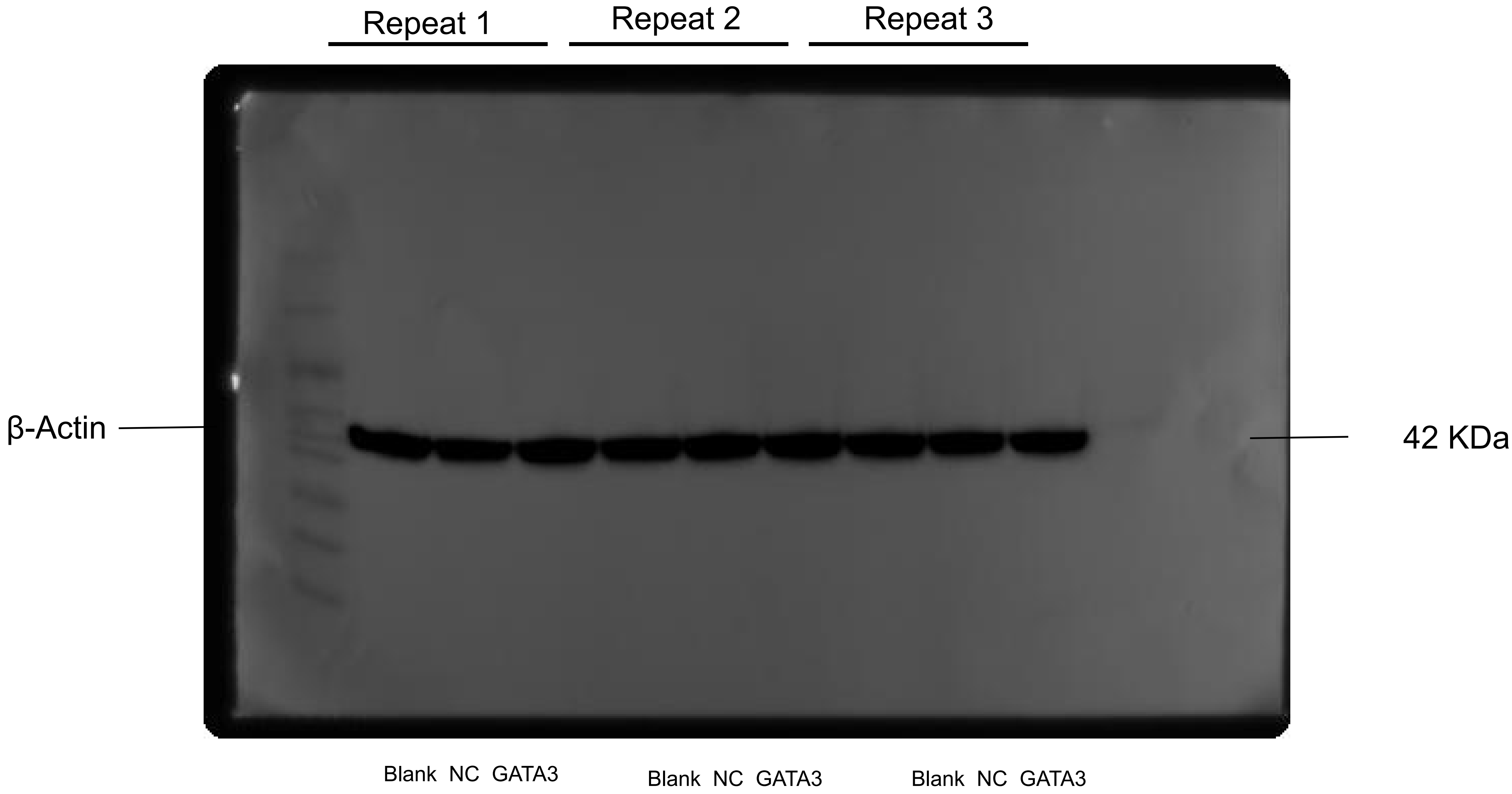
Repeat 1
Repeat 2
Repeat 3
β-Actin
42 KDa
Blank NC GATA3
Blank NC GATA3
Blank NC GATA3

Figure 4D

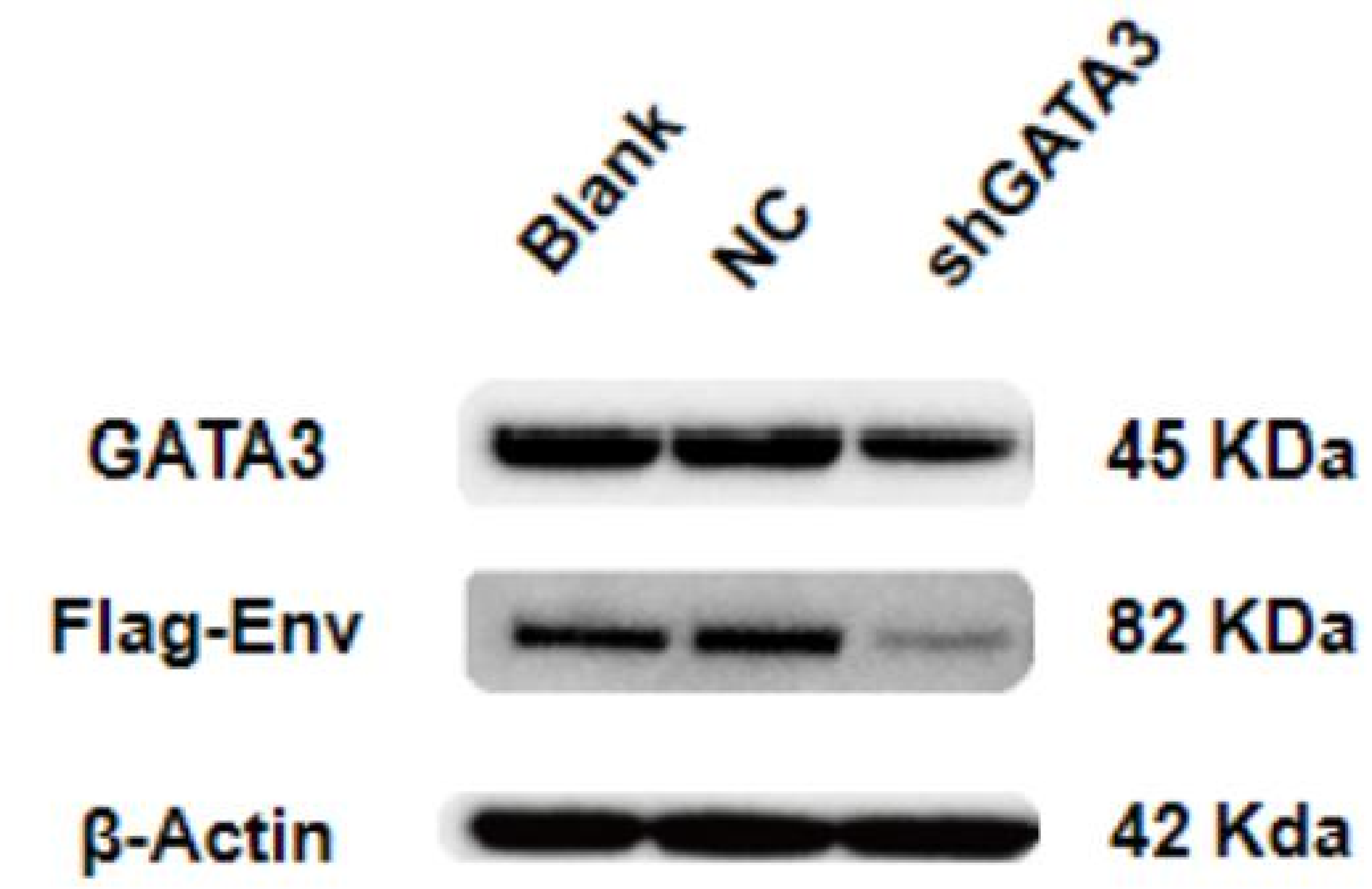

Figure 4D

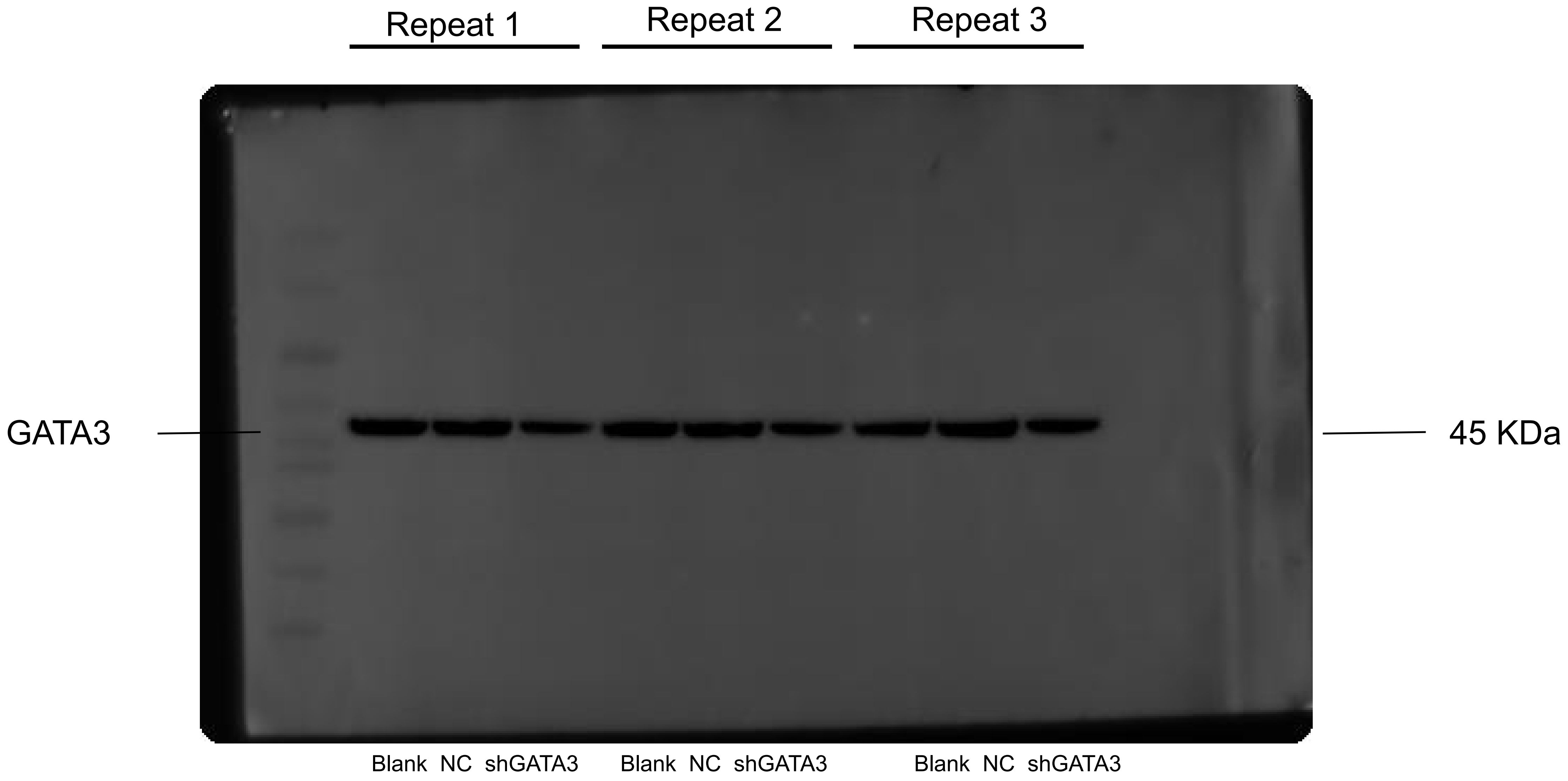
Repeat 1
Repeat 2
Repeat 3
GATA3
45 KDa
Blank NC shGATA3
Blank NC shGATA3
Blank NC shGATA3

Figure 4D

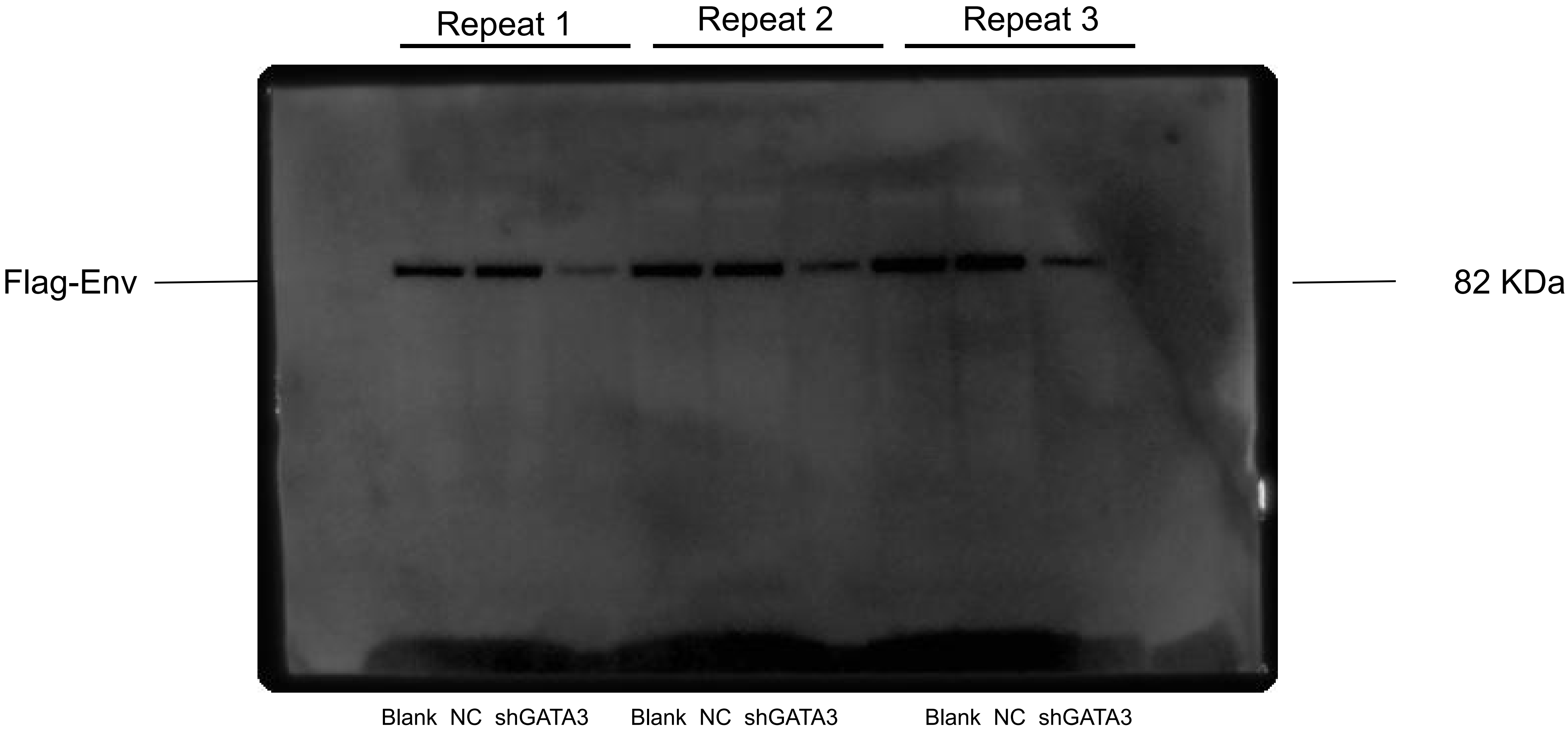

Repeat 1
Repeat 2
Repeat 3
Flag-Env
82 KDa
Blank NC shGATA3
Blank NC shGATA3
Blank NC shGATA3

Figure 4D

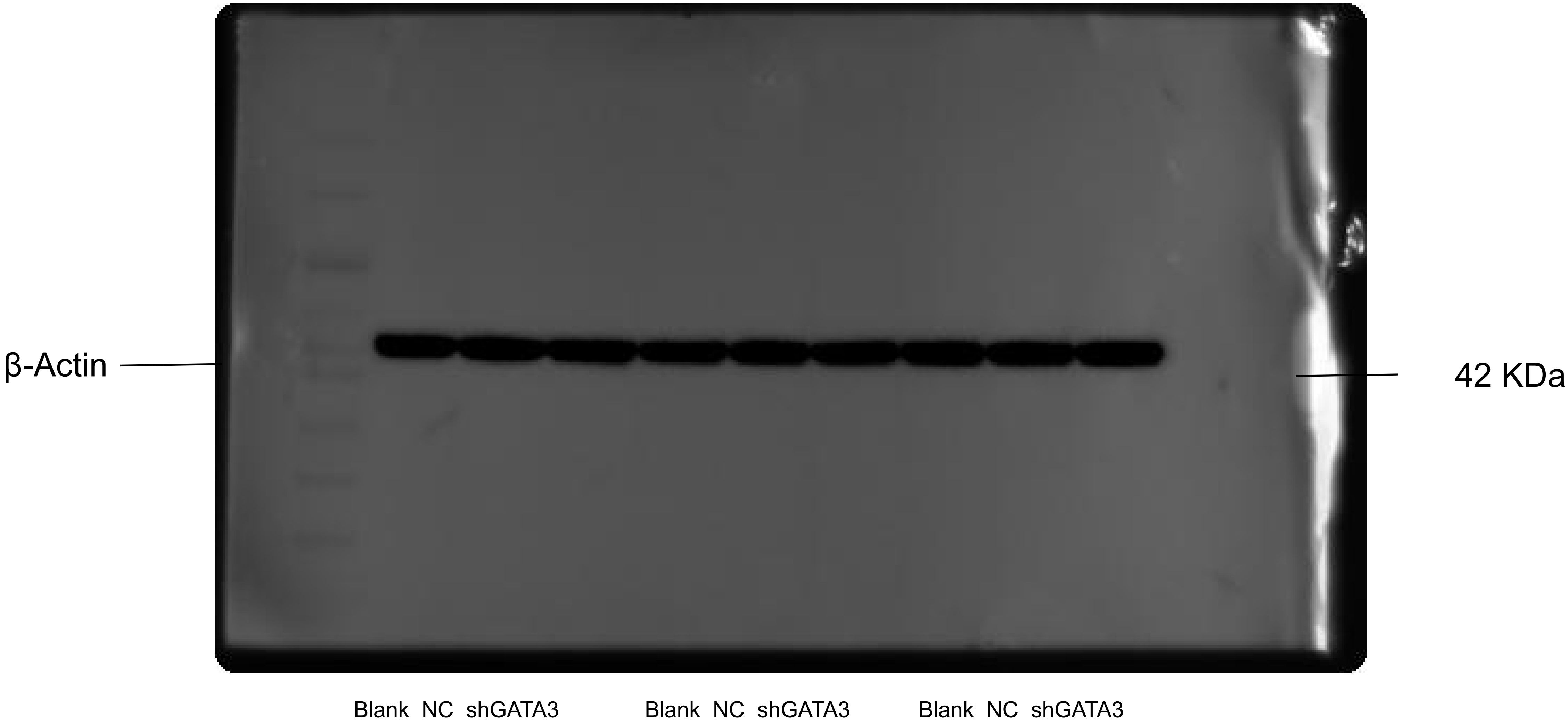
β-Actin
42 KDa
Blank NC shGATA3 Blank NC shGATA3 Blank NC shGATA3

Figure 3C

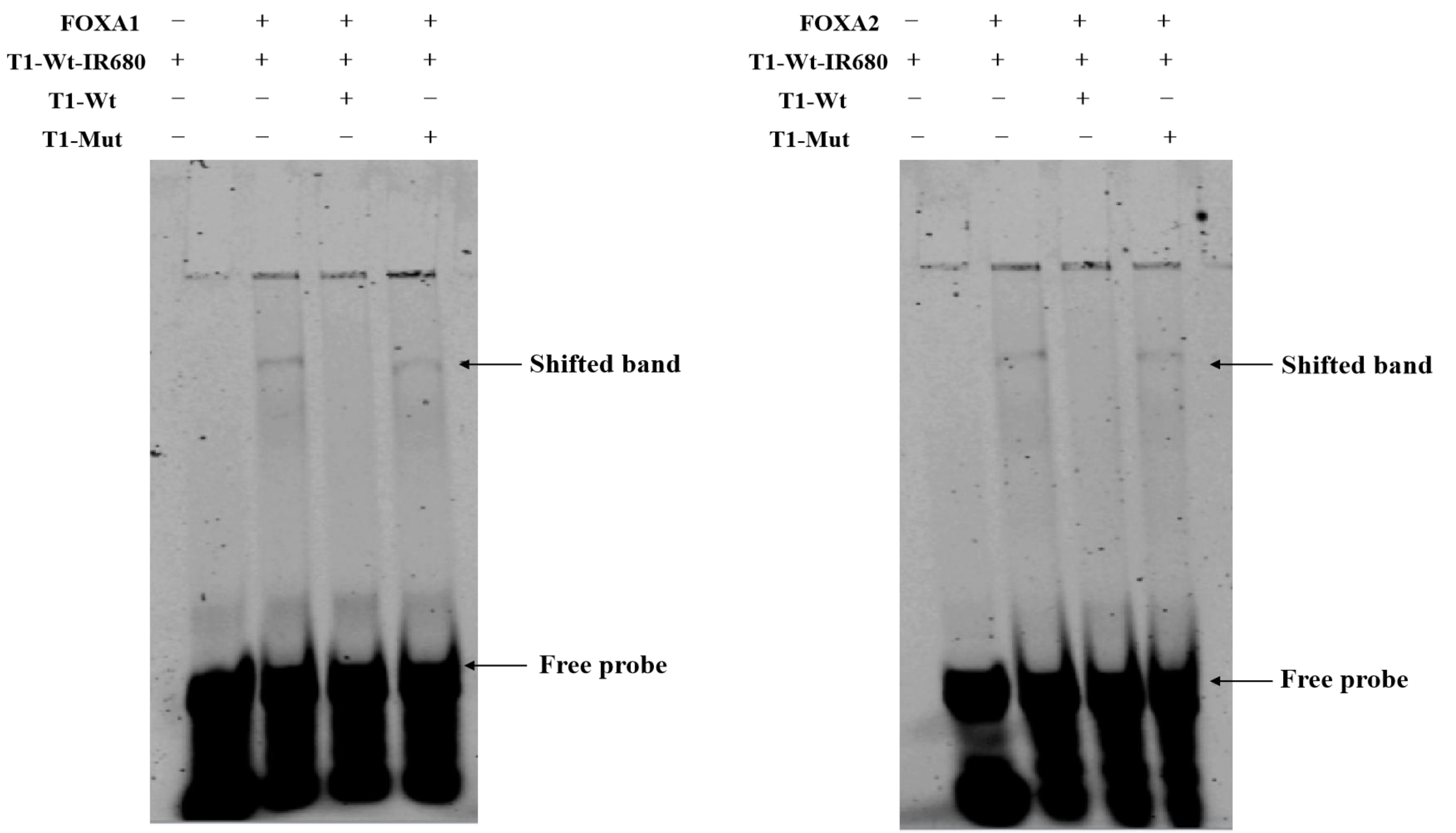
FOXA1 − + + +
T1-Wt-IR680 + + + +
T1-Wt − − + −
T1-Mut − − − +
Shifted band
Free probe
FOXA2 − + + +
T1-Wt-IR680 + + + +
T1-Wt − − + −
T1-Mut − − − +
Shifted band
Free probe

Figure 3C

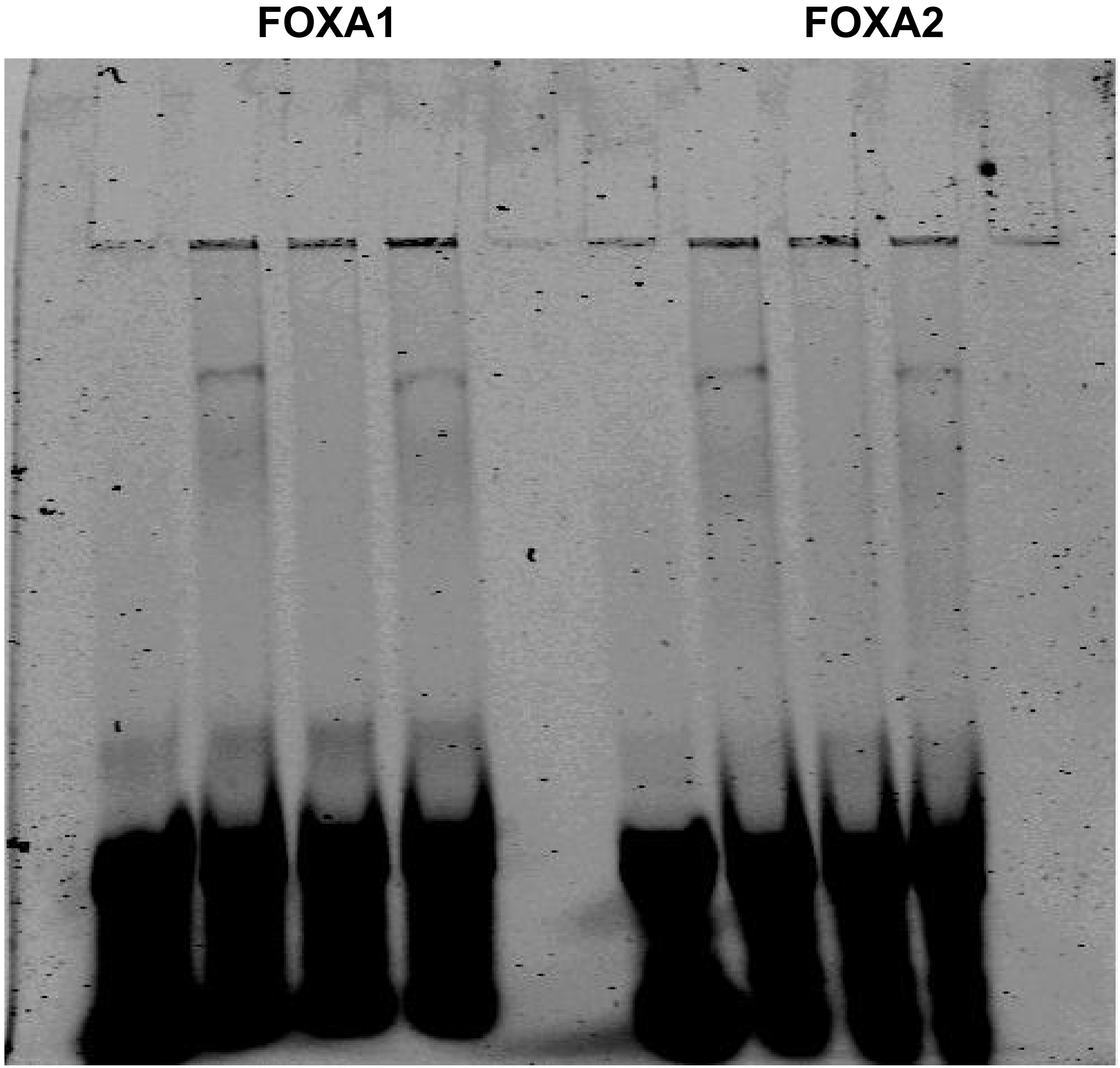

Figure 3C

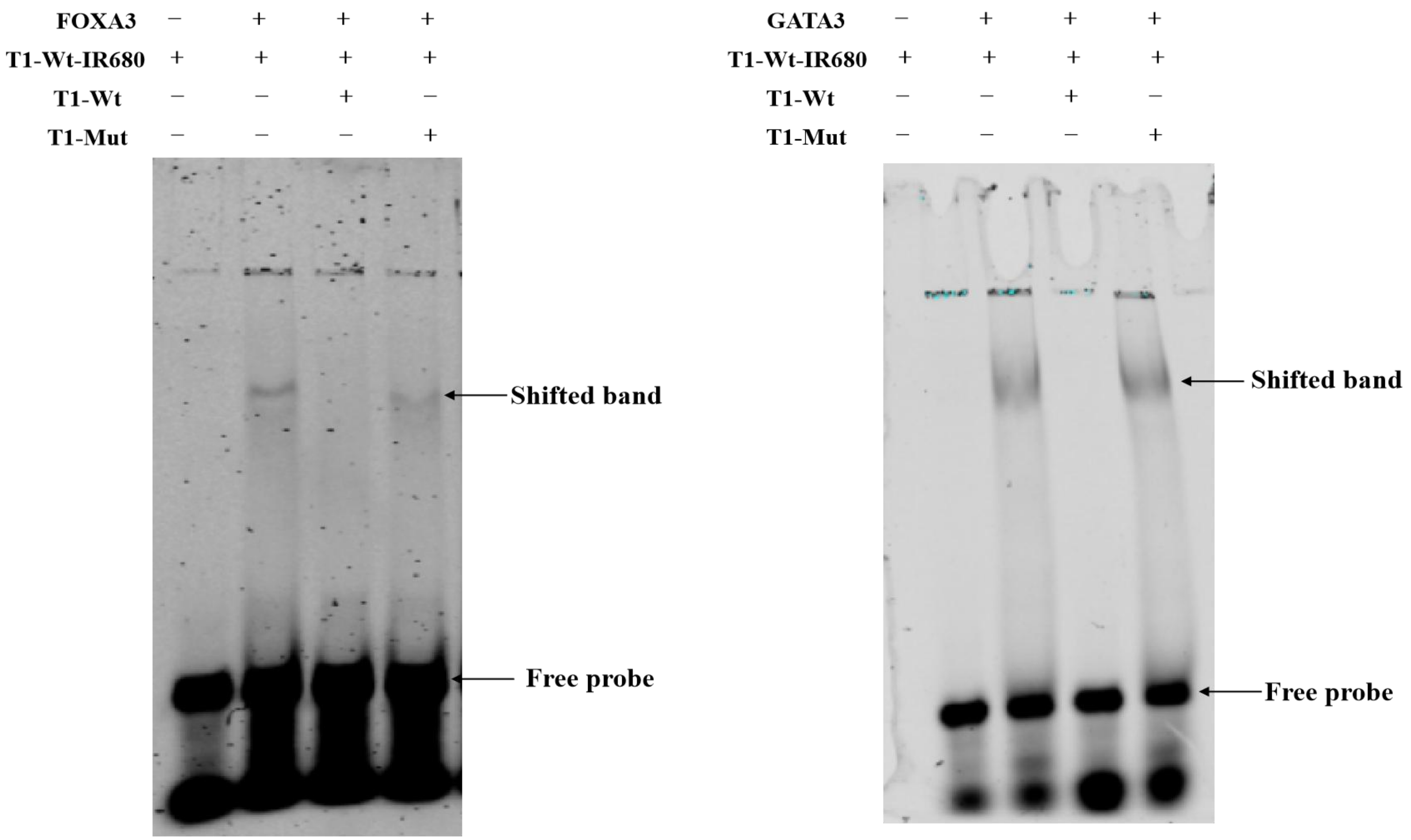

FOXA3 − + + +
T1-Wt-IR680 + + + +
T1-Wt − − + −
T1-Mut − − − +
Shifted band
Free probe
GATA3 − + + +
T1-Wt-IR680 + + + +
T1-Wt − − + −
T1-Mut − − − +
Shifted band
Free probe

Figure 3C

FOXA3

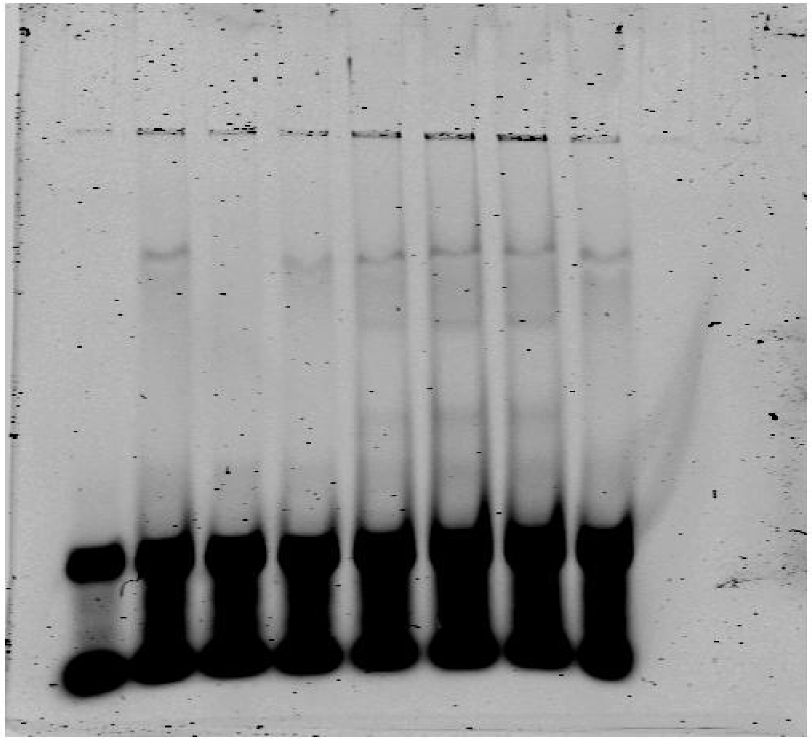

GATA3

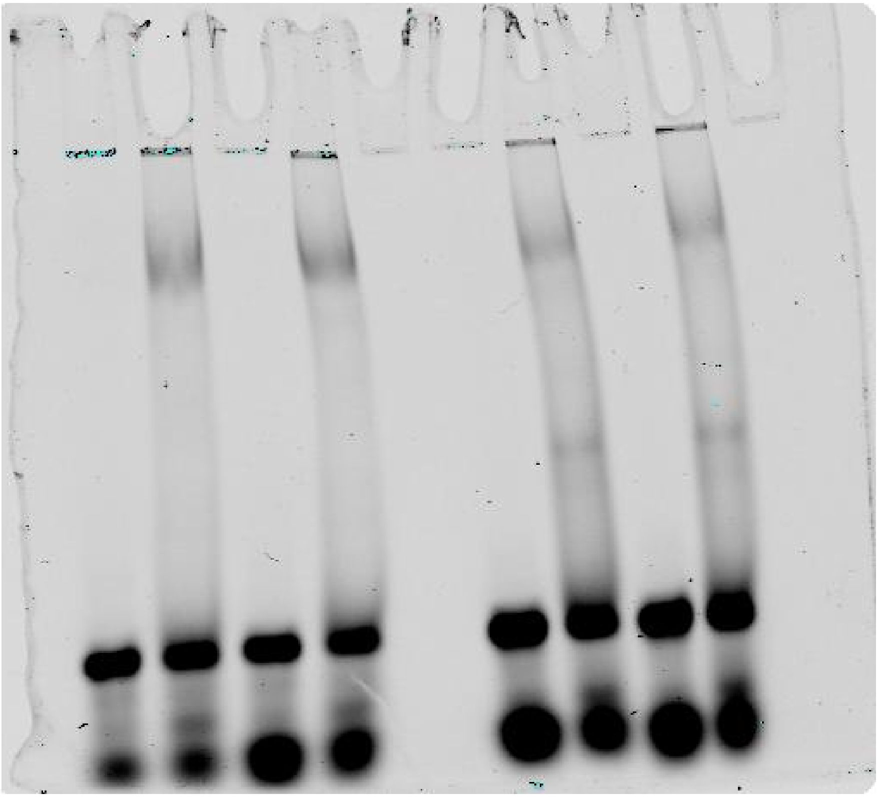

Supplement: Supplementary file 4 [file Data_Sheet_1.pdf]
