# Supplementary figures and images for: Investigation of exJSRV LTR promoter activity based on transcription factor regulatory networks
Source: Front Vet Sci. 2026 Jan 9;12:1727983. doi: 10.3389/fvets.2025.1727983 (PMC12827560; doi:10.3389/fvets.2025.1727983)

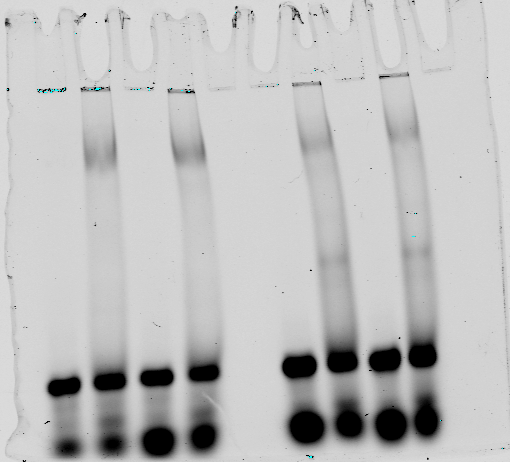

Supplement: Supplementary file 5 [file Data_Sheet_2.ZIP › original uncropped Western blot images/Figure3C/Figure 3C-GATA3.png]

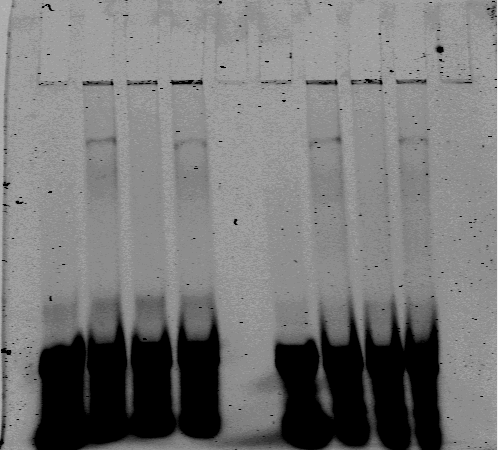

Supplement: Supplementary file 5 [file Data_Sheet_2.ZIP › original uncropped Western blot images/Figure3C/Figure 3C-FOXA1andFOXA2.png]

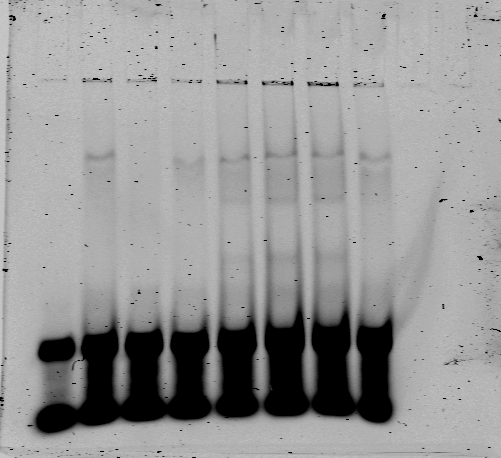

Supplement: Supplementary file 5 [file Data_Sheet_2.ZIP › original uncropped Western blot images/Figure3C/Figure 3C-FOXA3.png]

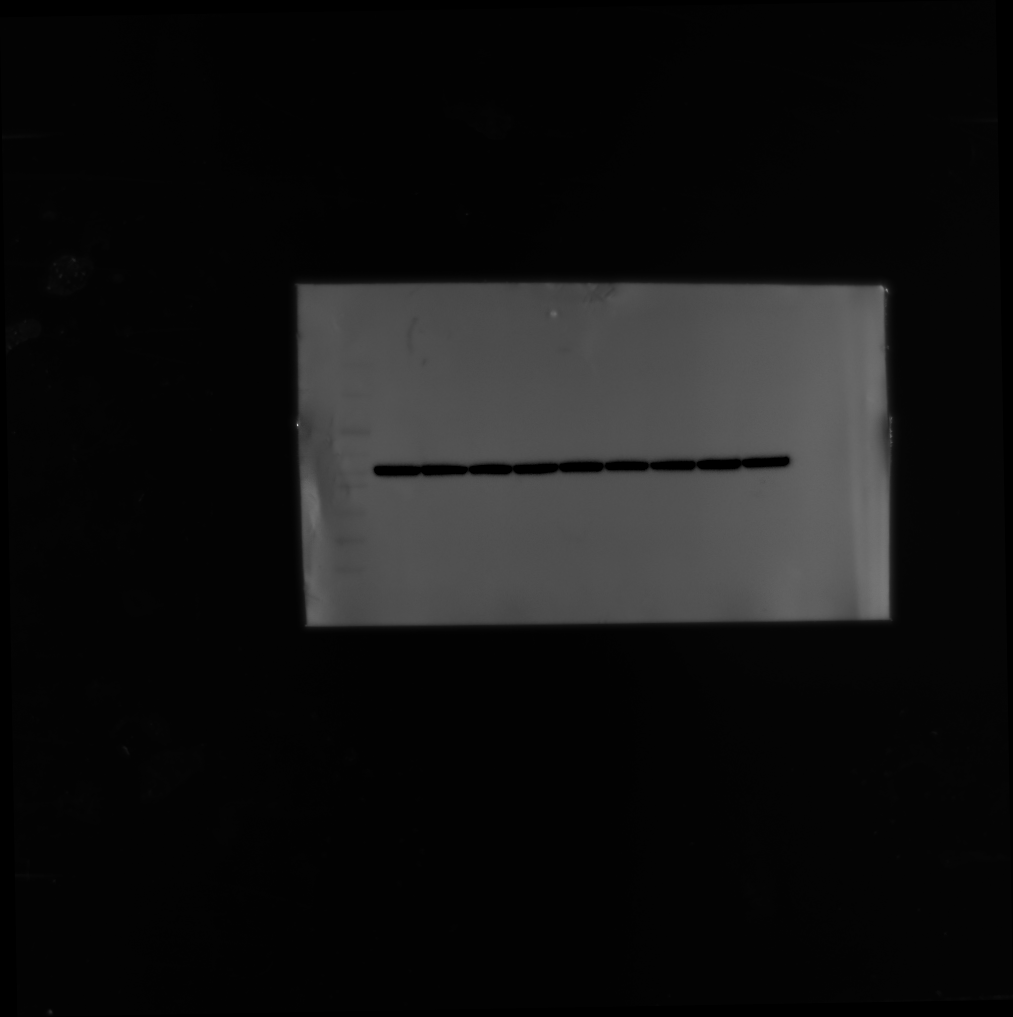

Supplement: Supplementary file 5 [file Data_Sheet_2.ZIP › original uncropped Western blot images/Figure4-C/Figure 4C-╬▓-actin 1-2.tiff]

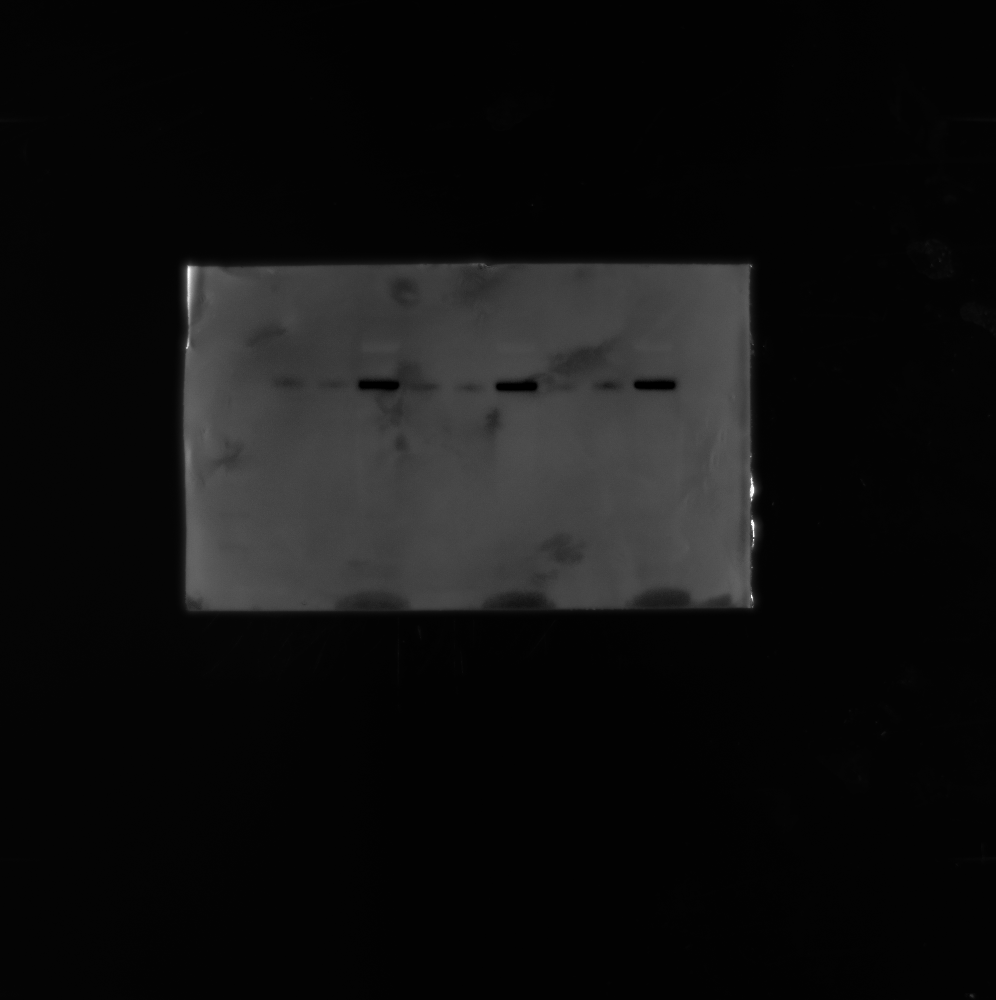

Supplement: Supplementary file 5 [file Data_Sheet_2.ZIP › original uncropped Western blot images/Figure4-C/Figure 4C-Flag-Env 1-2.tiff]

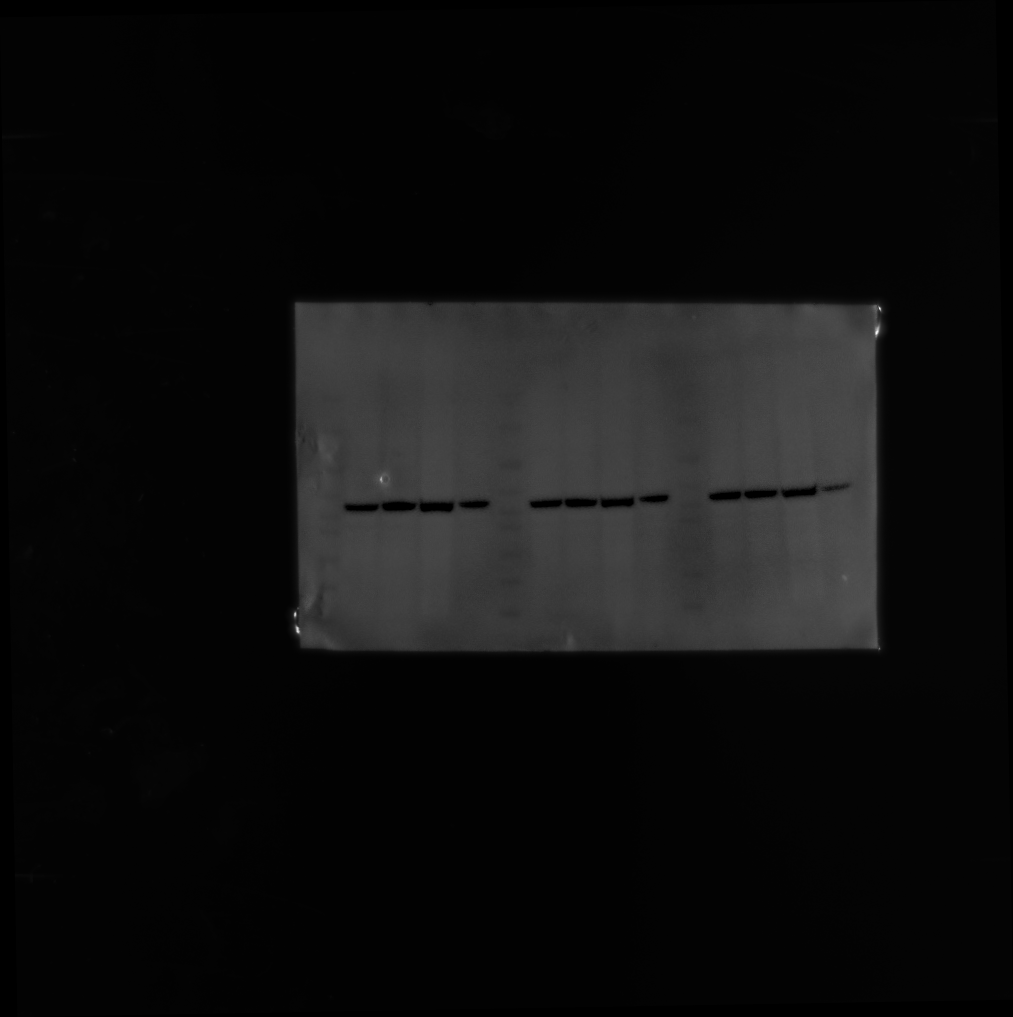

Supplement: Supplementary file 5 [file Data_Sheet_2.ZIP › original uncropped Western blot images/Figure2-B/Figure 2B-p-MEK 1-2.tiff]

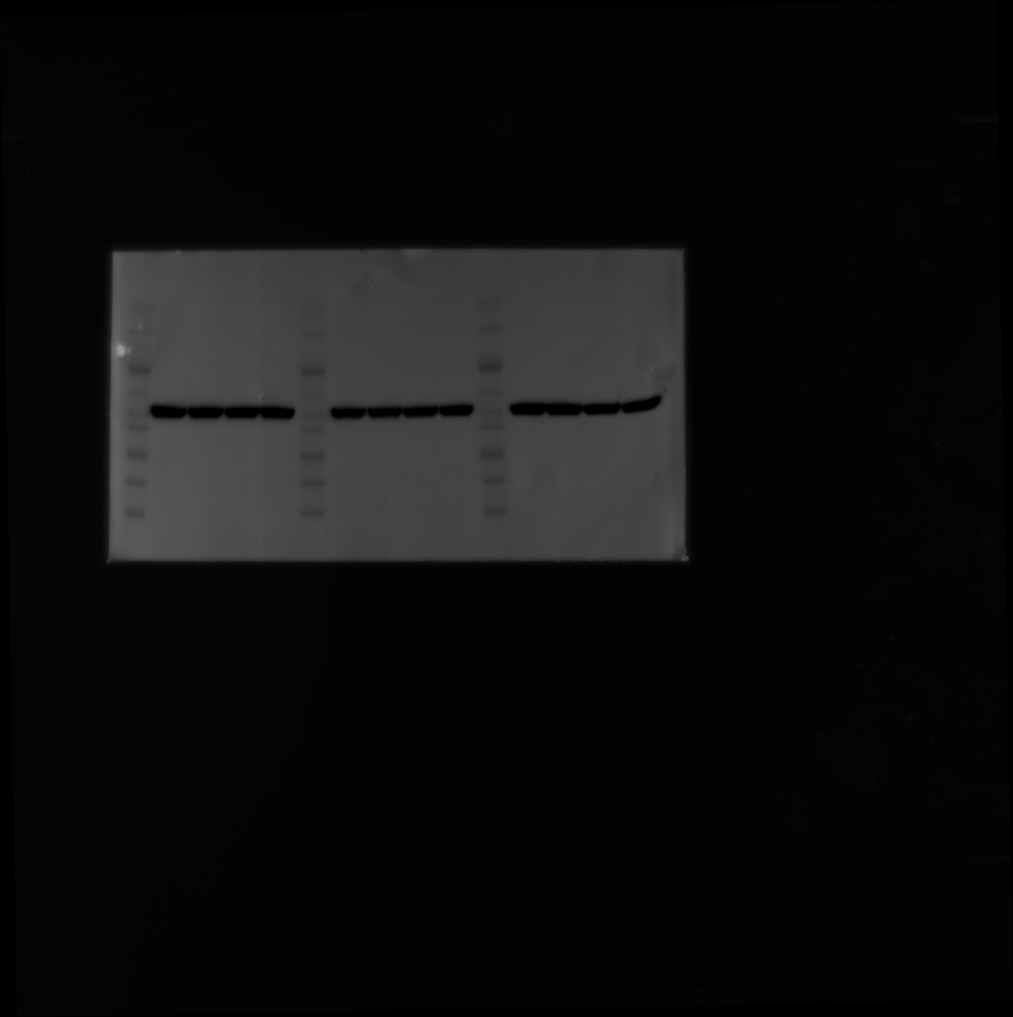

Supplement: Supplementary file 5 [file Data_Sheet_2.ZIP › original uncropped Western blot images/Figure2-B/Figure 2B-╬▓-actin 1-2.tiff]

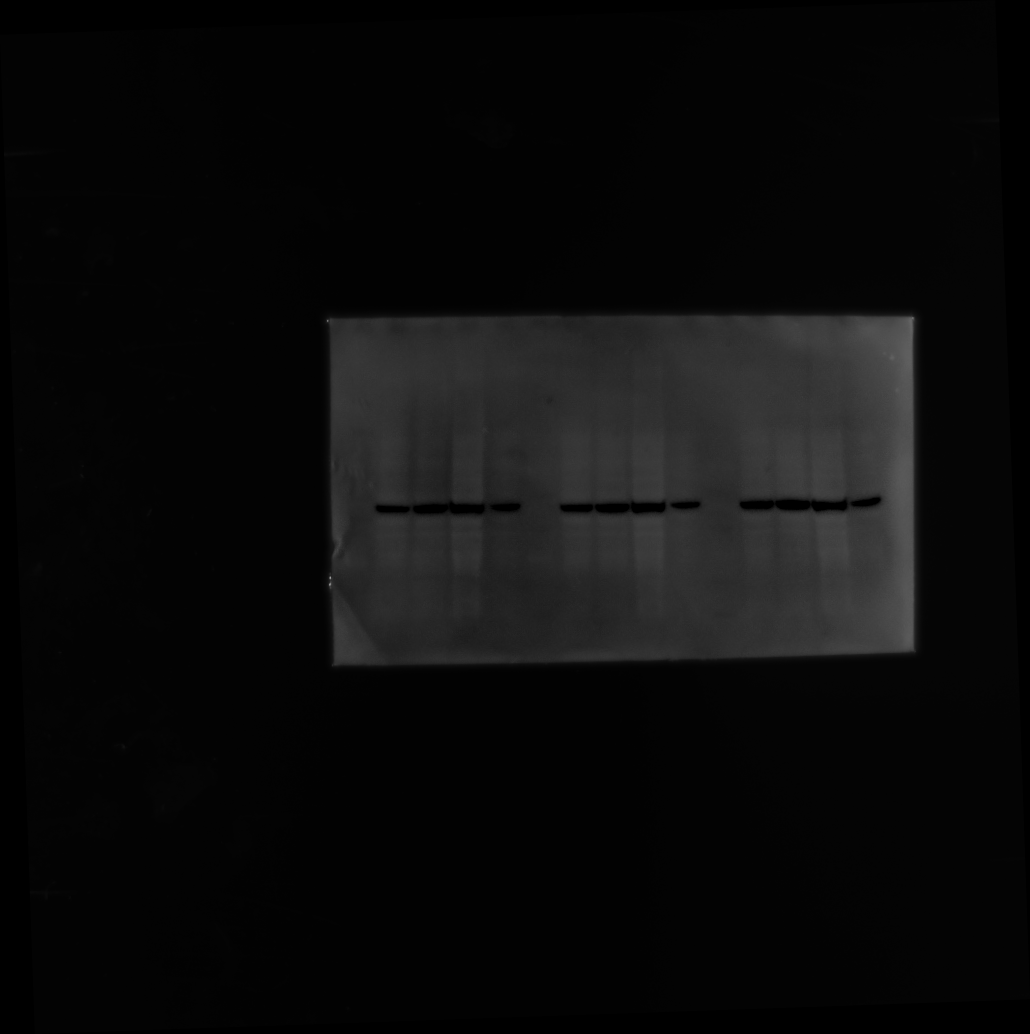

Supplement: Supplementary file 5 [file Data_Sheet_2.ZIP › original uncropped Western blot images/Figure2-B/Figure 2B-MEK 1-2.tiff]

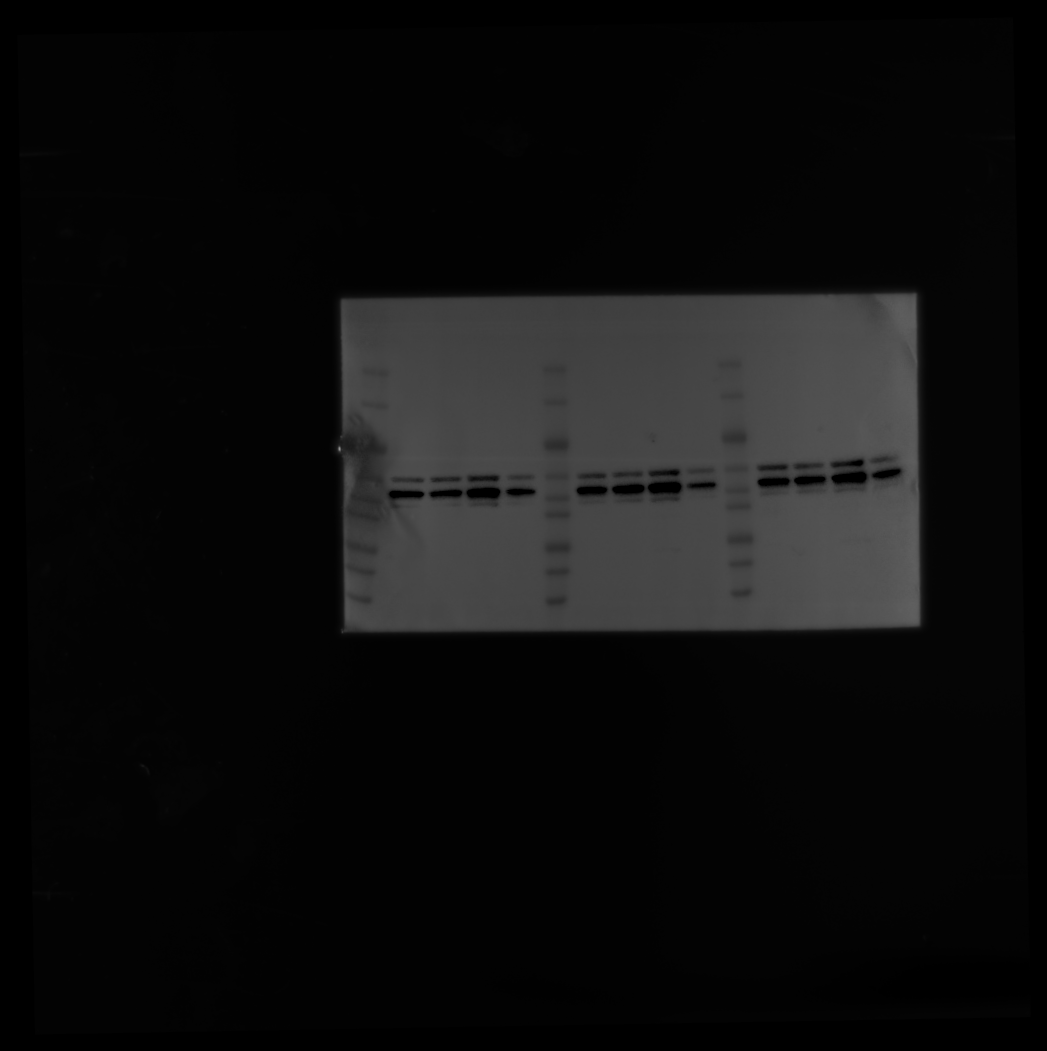

Supplement: Supplementary file 5 [file Data_Sheet_2.ZIP › original uncropped Western blot images/Figure2-C/Figure 2C-ERK 1-2.tiff]

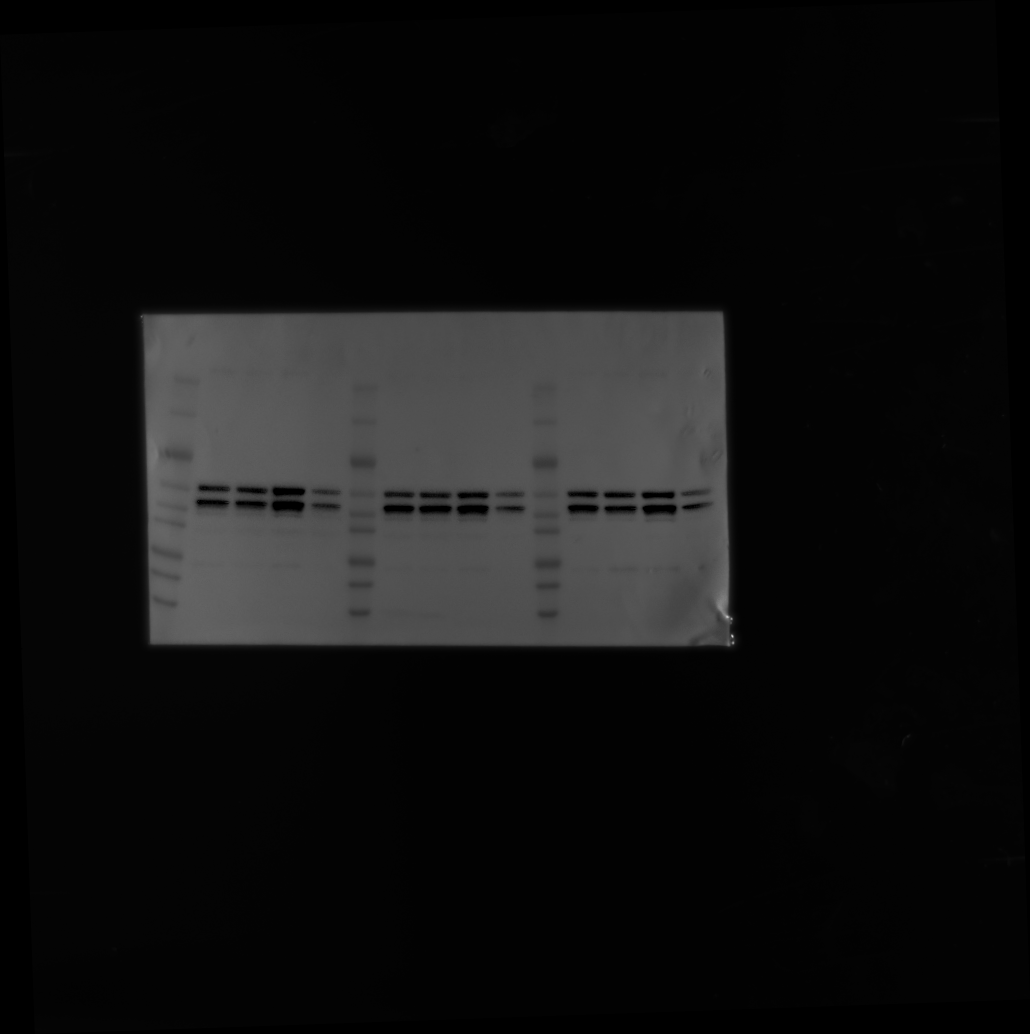

Supplement: Supplementary file 5 [file Data_Sheet_2.ZIP › original uncropped Western blot images/Figure2-C/Figure 2C-p-ERK 1-2.tiff]

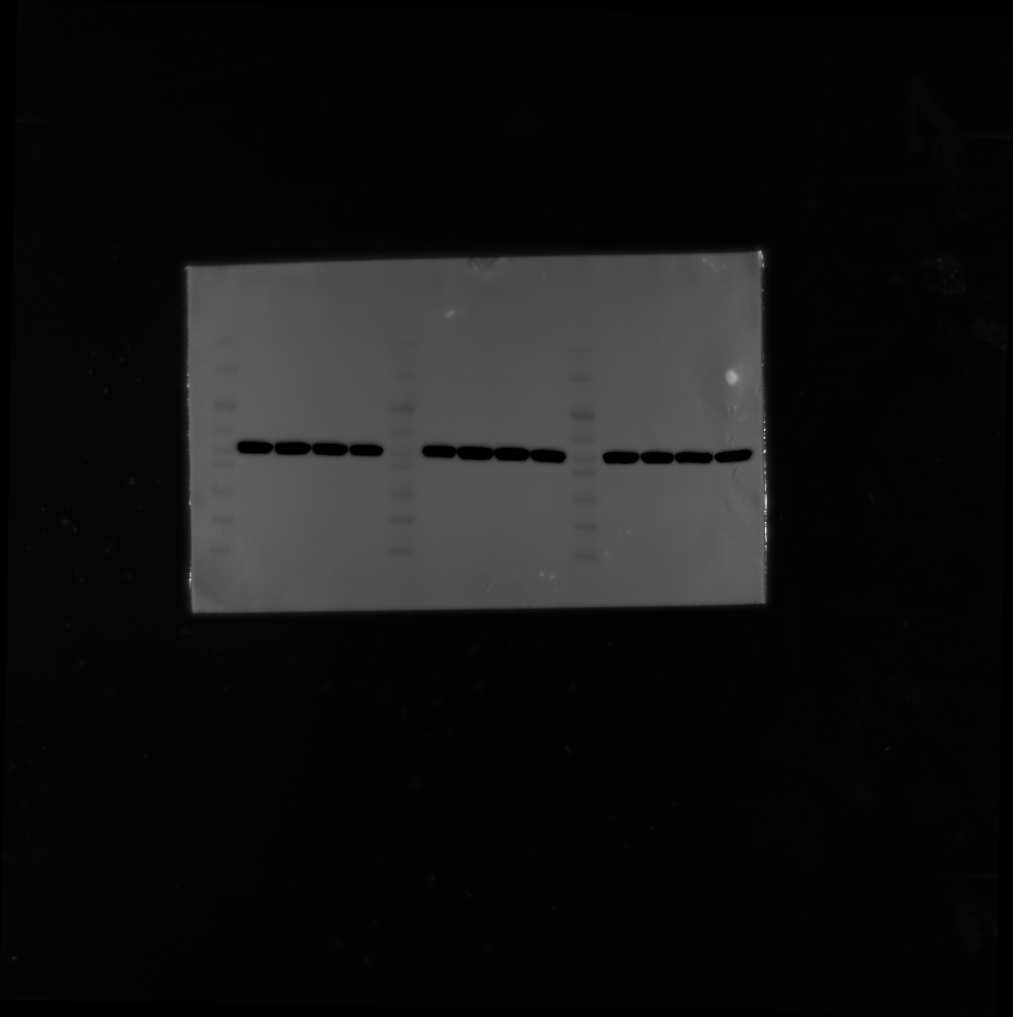

Supplement: Supplementary file 5 [file Data_Sheet_2.ZIP › original uncropped Western blot images/Figure2-C/Figure 2C-╬▓-actin 1-2.tiff]

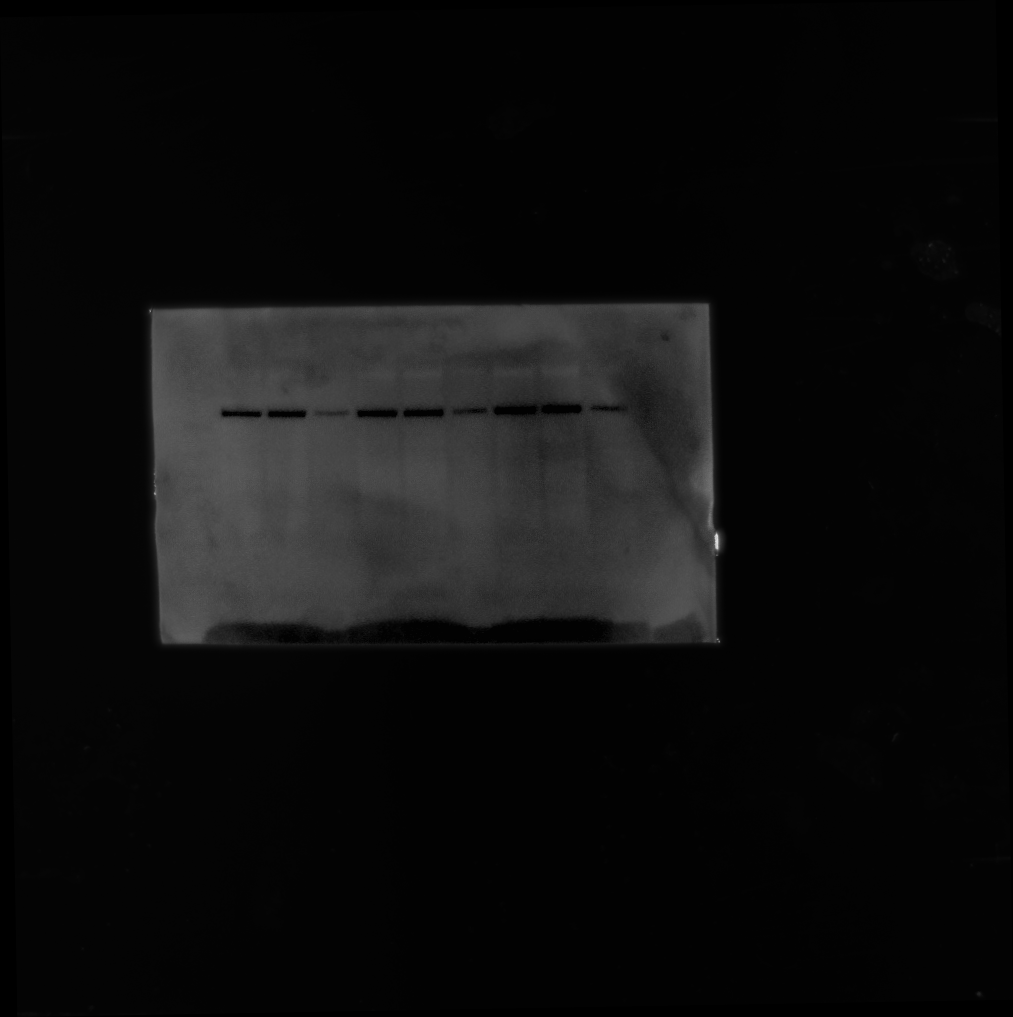

Supplement: Supplementary file 5 [file Data_Sheet_2.ZIP › original uncropped Western blot images/Figure4-D/shGATA3/Figure 4D-shGATA3-Flag-Env 1-2.tiff]

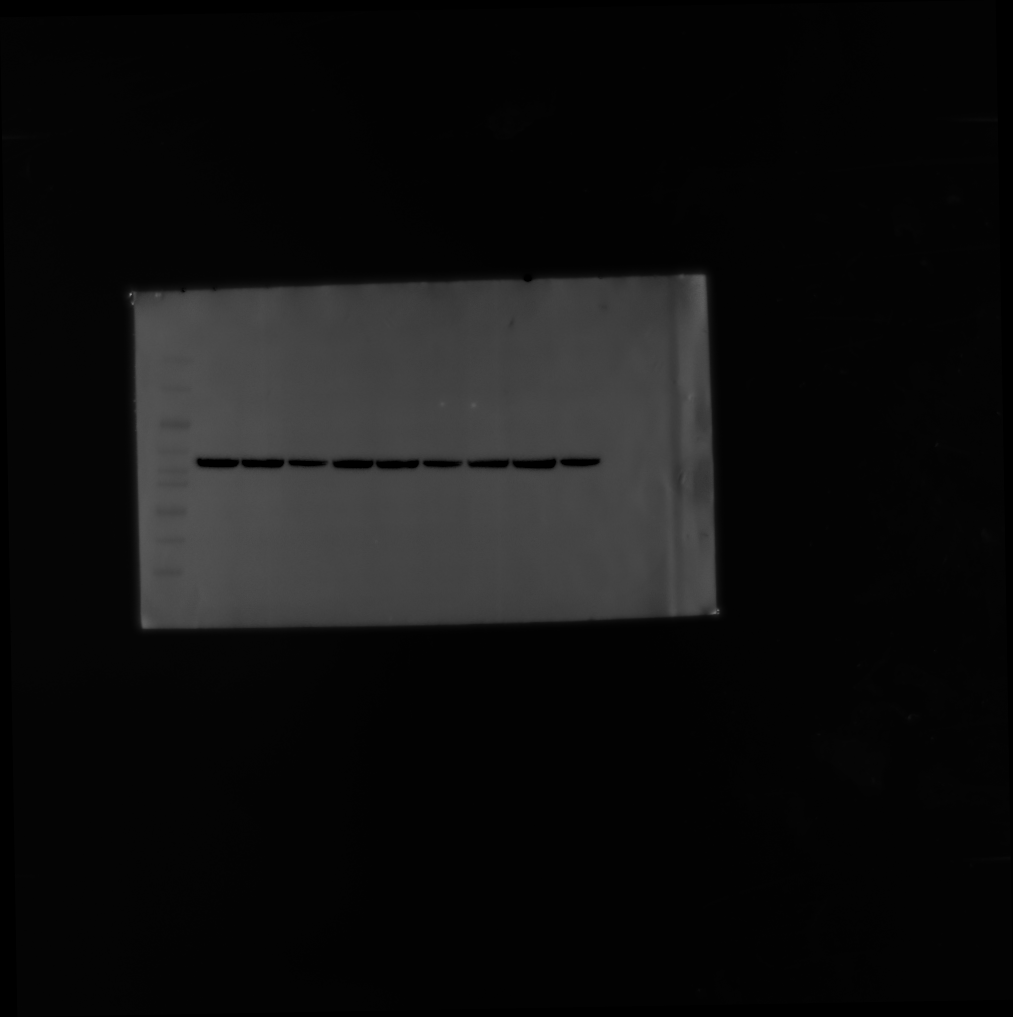

Supplement: Supplementary file 5 [file Data_Sheet_2.ZIP › original uncropped Western blot images/Figure4-D/shGATA3/Figure 4D-shGATA3-GATA3 1-2.tiff]

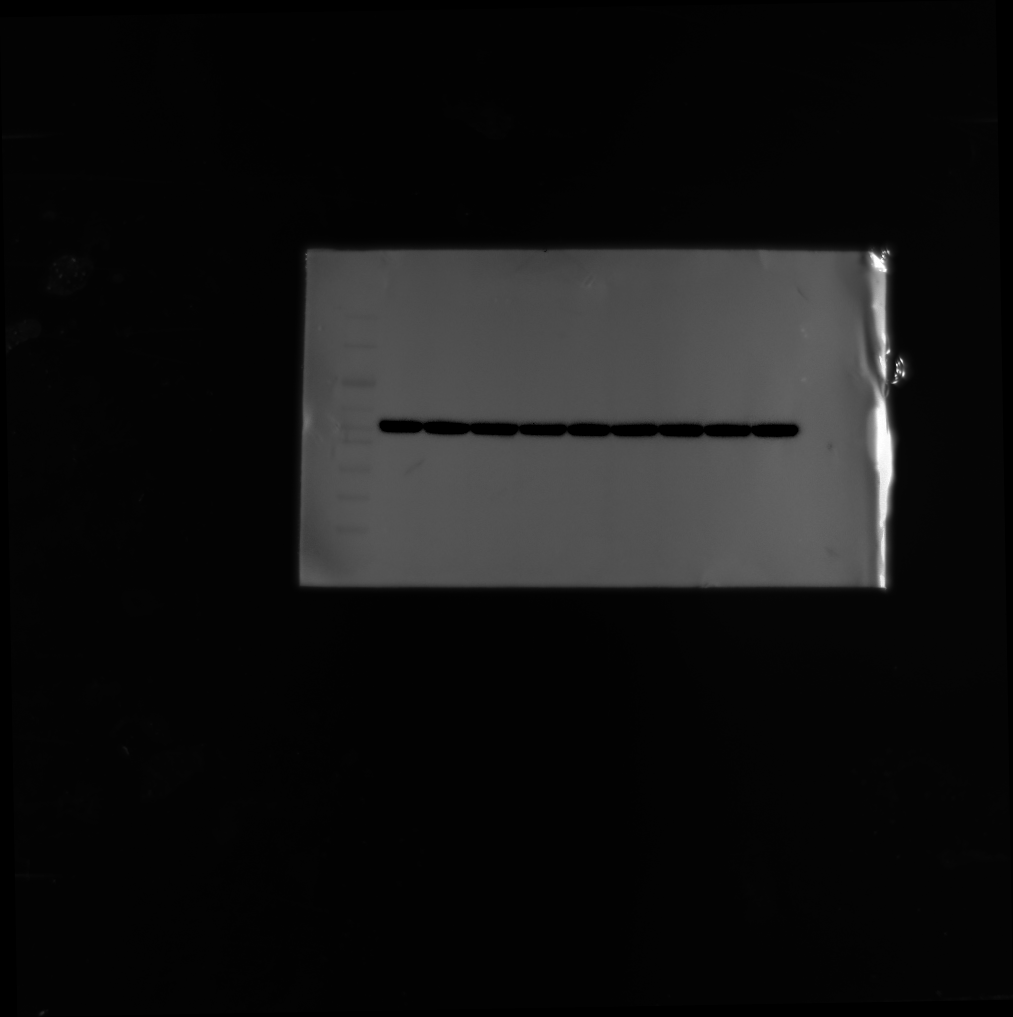

Supplement: Supplementary file 5 [file Data_Sheet_2.ZIP › original uncropped Western blot images/Figure4-D/shGATA3/Figure 4D-shGATA3-╬▓-actin 1-2.tiff]

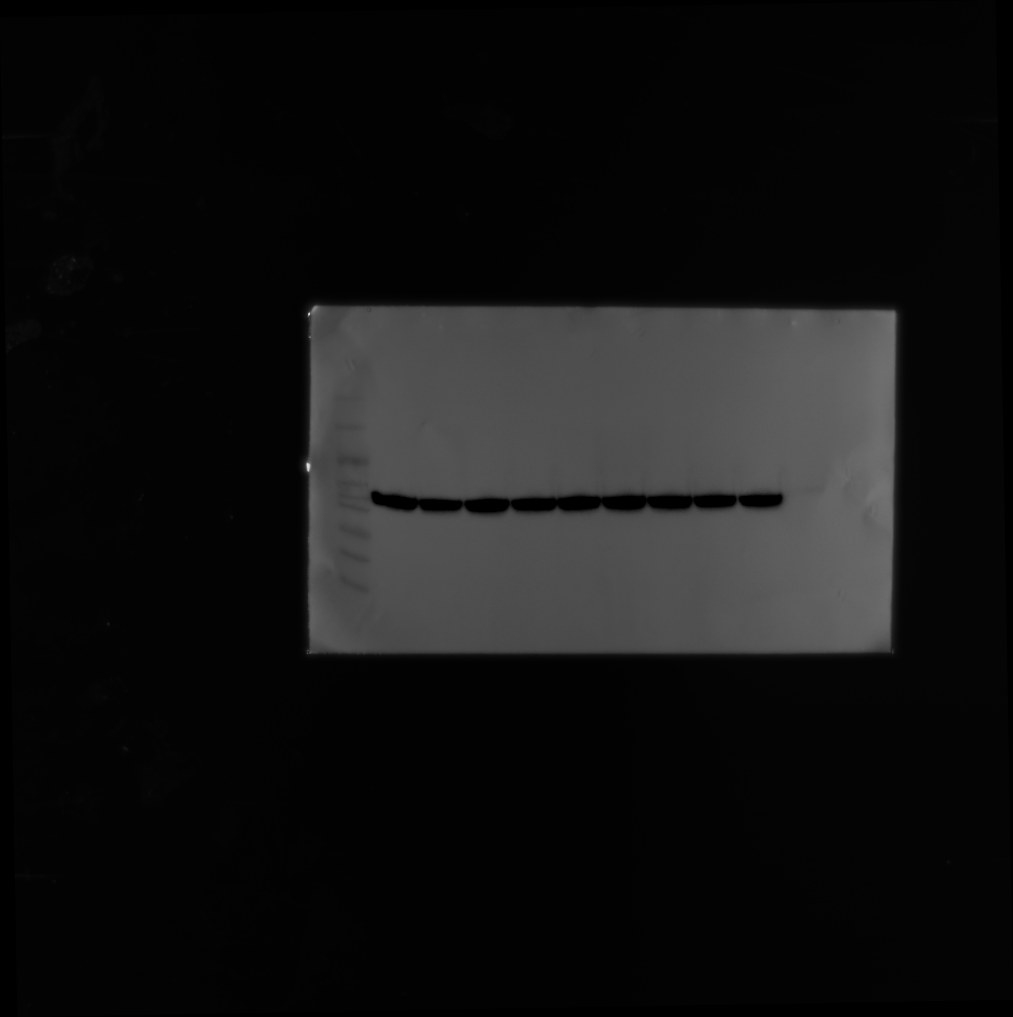

Supplement: Supplementary file 5 [file Data_Sheet_2.ZIP › original uncropped Western blot images/Figure4-D/GATA3/Figure 4D-GATA3-╬▓-actin 1-2.tiff]

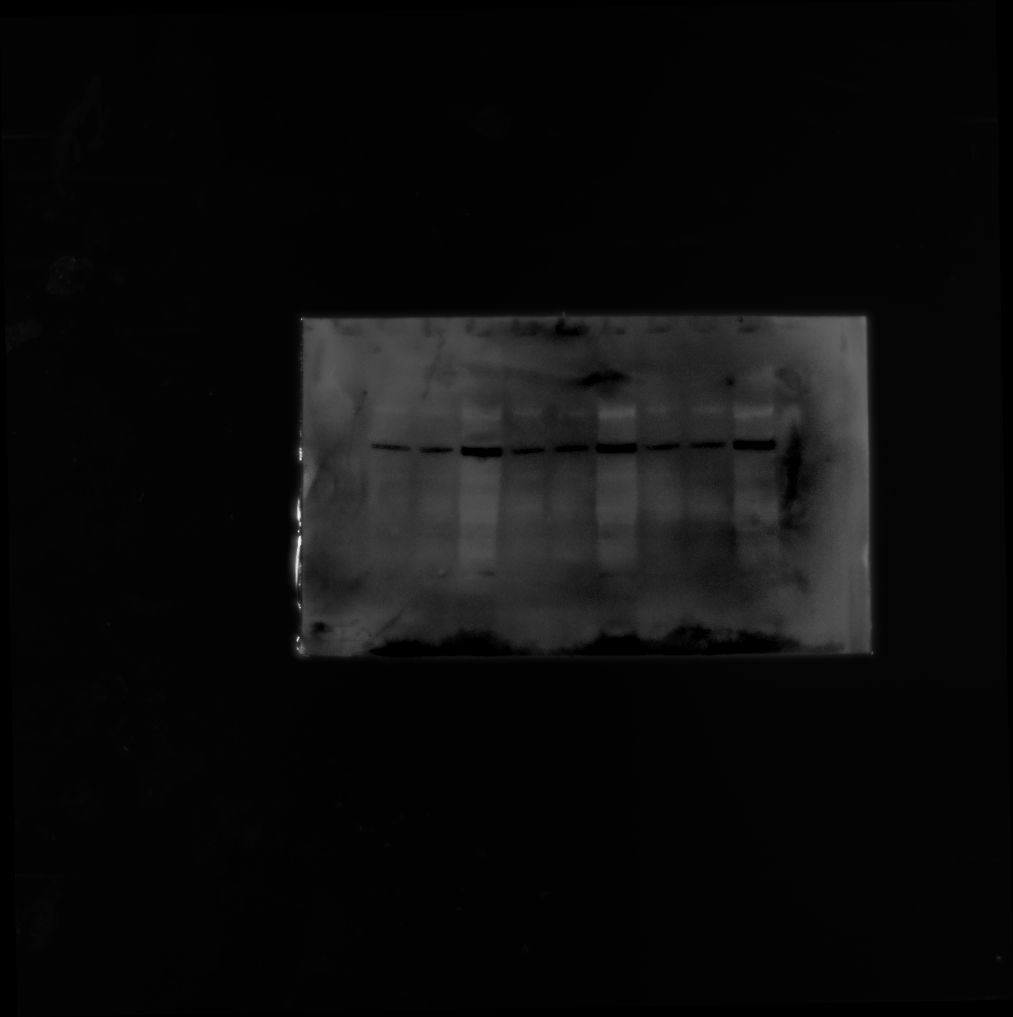

Supplement: Supplementary file 5 [file Data_Sheet_2.ZIP › original uncropped Western blot images/Figure4-D/GATA3/Figure 4D-GATA3-Flag-Env 1-2.tiff]

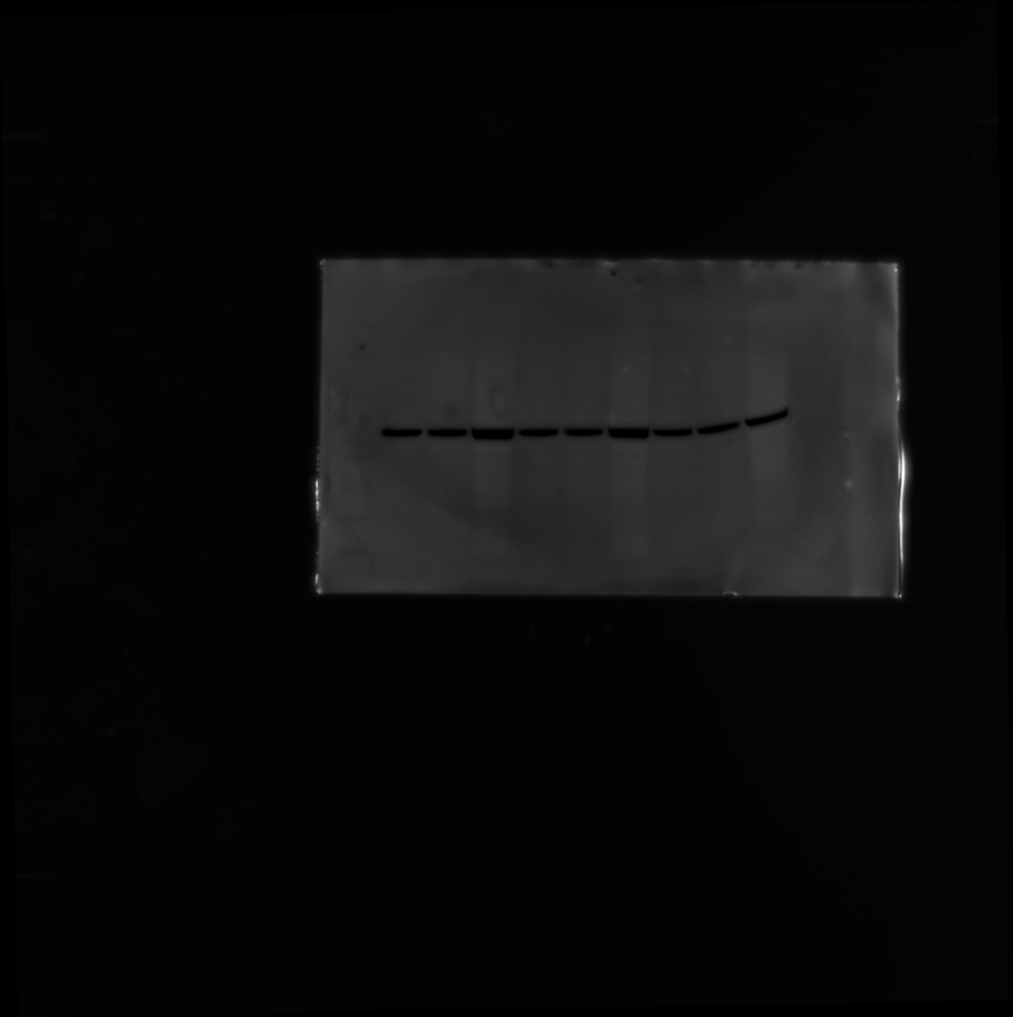

Supplement: Supplementary file 5 [file Data_Sheet_2.ZIP › original uncropped Western blot images/Figure4-D/GATA3/Figure 4D-GATA3-GATA3 1-2.tiff]
